# The PavDREB1E–PavD8–PavHY5 module integrates light and gibberellin signals to regulate sweet cherry anthocyanin biosynthesis

**DOI:** 10.1093/plphys/kiaf616

**Published:** 2025-11-26

**Authors:** Yuqin Xiao, Xiang Zhang, Bingyang Du, Maihemuti Turupu, Qiaoqiao Zhang, Qisheng Yao, Xinyu Wang, Zhengyang Wen, Shuo Wang, Wenjun Lu, Tianhong Li

**Affiliations:** Frontiers Science Center for Molecular Design Breeding, College of Horticulture, China Agricultural University, Beijing 100193, China; Frontiers Science Center for Molecular Design Breeding, College of Horticulture, China Agricultural University, Beijing 100193, China; Frontiers Science Center for Molecular Design Breeding, College of Horticulture, China Agricultural University, Beijing 100193, China; Frontiers Science Center for Molecular Design Breeding, College of Horticulture, China Agricultural University, Beijing 100193, China; Frontiers Science Center for Molecular Design Breeding, College of Horticulture, China Agricultural University, Beijing 100193, China; Frontiers Science Center for Molecular Design Breeding, College of Horticulture, China Agricultural University, Beijing 100193, China; Frontiers Science Center for Molecular Design Breeding, College of Horticulture, China Agricultural University, Beijing 100193, China; Frontiers Science Center for Molecular Design Breeding, College of Horticulture, China Agricultural University, Beijing 100193, China; Frontiers Science Center for Molecular Design Breeding, College of Horticulture, China Agricultural University, Beijing 100193, China; Frontiers Science Center for Molecular Design Breeding, College of Horticulture, China Agricultural University, Beijing 100193, China; Frontiers Science Center for Molecular Design Breeding, College of Horticulture, China Agricultural University, Beijing 100193, China

## Abstract

Light stimulates anthocyanin accumulation in bicolored sweet cherry (*Prunus avium* cv. Rainier) fruits, resulting in red pigmentation. The phytohormone gibberellin (GA) plays an important role in fruit coloration, but the molecular mechanisms that integrate light and GA signaling pathways during anthocyanin biosynthesis remain poorly understood. We show that light induces anthocyanin accumulation but reduces the gibberellic acid (GA_3_) content in “Rainier” sweet cherry fruits and that GA_3_ treatment inhibits light-induced anthocyanin accumulation. Light exposure and low GA_3_ levels induce the transcription of a DEHYDRATION RESPONSIVE ELEMENT-BINDING PROTEIN (DREB) gene, *PavDREB1E*, which encodes a protein that directly activates the transcription of key anthocyanin biosynthesis genes, including *chalcone synthase* (*PavCHS*), *dihydroflavonol-4-reductase* (*PavDFR*), and *UDP glucose-flavonoid-3-O-glycosyltransferase* (*PavUFGT*). The DELLA protein PavDWARF8 (PavD8), which is stabilized under low GA_3_ levels, interacts with PavDREB1E to strengthen its transcriptional activation activity. Furthermore, light-induced ELONGATED HYPOCOTYL5 (PavHY5) promotes this interaction. In the dark, *PavDREB1E* and *PavHY5* transcript levels are low. Moreover, PavDREB1E, PavD8, and PavHY5 are degraded in the dark and accumulate in the light via the light-induced nuclear depletion of the E3 ubiquitin ligases CONSTITUTIVELY PHOTOMORPHOGENIC 1 (PavCOP1-1 and PavCOP1-2). We present a mechanism by which the PavDREB1E–PavD8–PavHY5 module integrates light and GA signaling to regulate anthocyanin biosynthesis in sweet cherry fruits.

## Teaser

The transcription factor PavDREB1E plays a key role in regulating sweet cherry fruit coloration by integrating light and gibberellin signals.

## Introduction

Sweet cherry (*Prunus avium* L.) is a widely popular fruit known for its attractive appearance, delicious taste, and rich nutrients (Jin et al. 2016; Karagiannis et al. 2021). Anthocyanins determine the appearance of sweet cherry fruits, which directly influences their market value, so improvement of anthocyanins accumulation is an important issue in fruit production (Esti et al. 2002; Liu et al. 2011). Anthocyanin biosynthesis depends on a series of enzymes, including phenylalanine ammonia lyase (PAL), chalcone synthase (CHS), chalcone isomerase (CHI), flavanone-3-β-hydroxylase (F3H), dihydroflavonol-4-reductase (DFR), anthocyanidin synthase (ANS), and UDP Glc-flavonoid-3-O glucosyltransferase (UFGT). The MBW complex, which consists of a MYB transcription factor, a basic helix–loop–helix (bHLH) transcription factor, and a WD-repeat protein, is the primary transcriptional regulator of anthocyanin accumulation (Espley et al. 2007; Allan and Espley 2018).

Anthocyanin accumulation is regulated by both internal and external factors, including light, temperature, plant hormones, sugar levels, and drought (Ohlsson and Berglund 2001; Wang et al. 2018; Yang et al. 2019; Peng et al. 2020; Ni et al. 2023). Among these, light plays a key role in anthocyanins biosynthesis. Abundant studies have revealed that light stimulates the production of anthocyanins, especially within the fruit skin. In many plants, such as tomato (*Solanum lycopersicum* Indigo Rose), eggplant (*Solanum melongena*), red Chinese sand pears (*Pyrus pyrifolia* Nakai), apple (*Malus domestica*), and bicolored sweet cherries, anthocyanins do not accumulate when the fruits are bagged, but they accumulate rapidly after debagging and exposure to light (Takos et al. 2006; Huang et al. 2009 ; Feng et al. 2013, 2014; Guo et al. 2018; Yang et al. 2019; Sun et al. 2020; Hu et al. 2021).

The CONSTITUTIVELY PHOTOMORPHOGENIC 1 (COP1)–ELONGATED HYPOCOTYL5 (HY5) pathway plays a pivotal role in light-mediated coloration. HY5, a basic leucine zipper (bZIP) transcription factor, increases anthocyanin content by directly binding to the promoters of anthocyanin biosynthetic structural genes and by interacting with other transcription factors, including HY5 HOMOLOG (HYH) and members of the BBX family, to activate the expression of anthocyanin biosynthesis genes (Lee et al. 2007; Shin et al. 2013; An et al. 2019; Bai et al. 2019a, 2019b; Bhatnagar et al. 2020). COP1, an E3 ubiquitin ligase, interacts with and mediates the ubiquitination of HY5, MYBs, and other positive regulators of anthocyanin biosynthesis in the nucleus, leading to their proteasomal degradation (An et al. 2019; Bhatnagar et al. 2020; Tao et al. 2020). More COP1 localizes to the nucleus in the dark vs. light, as light-induced COP1 translocates to the cytosol (von Arnim and Deng 1994). Although there has been some research focusing on light-induced anthocyanin accumulation in plants, additional factors involved in this biological process remain unidentified.

In numerous plant species such as petunia (*Petunia hybrida*), hyacinth (*Hyacinthus orientalis*), Arabidopsis (*Arabidopsis thaliana*), apple, pear (*Pyrus bretschneideri*), litchi (*Litchi chinensis*), and sweet cherry, the role of gibberellin (GA) in regulating anthocyanin biosynthesis has been documented (Weiss et al. 1995; Hosokawa 1999; Choi et al. 2002; Loreti et al. 2008; Zhang et al. 2011; Zhai et al. 2019; Li et al. 2020; Kuhn et al. 2021; Qu et al. 2022; An et al. 2024a, 2024b). Particularly, Kuhn et al. reported that GA_3_ changed the expression of ABA biosynthetic and response genes in the early-season sweet cherry variety, suggesting GA_3_ may affect anthocyanin through reducing ABA levels (Kuhn et al. 2021). DELLA, a negative regulator of GA signaling, positively modulates anthocyanin biosynthesis by indirectly inhibiting repressors of the MBW complex and directly enhancing MBW complex activity through protein–protein interactions (Li et al. 2014; Qi et al. 2014 ; Xie et al. 2016 ; An et al. 2023; You et al. 2023). When GA levels are high, GA mediates the interactions of DELLAs with the SCF (SKP1-CUL1-F-box protein) E3 ligase complex in Arabidopsis, leading to DELLA degradation via the 26S proteasome and eventually triggering GA signaling. Conversely, under low-GA conditions, DELLAs accumulate and inhibit the GA signaling pathway (Murase et al. 2008; Davière and Achard 2013). Furthermore, anthocyanin biosynthesis is synergistically regulated by light and various plant hormones, including jasmonate, ethylene, brassinosteroid, and abscisic acid (Liu et al. 2015; An et al. 2017; Samkumar et al. 2021; Wang et al. 2023a, 2023b; Li et al. 2024; Zhang et al. 2024). There are researches on the effect of GA_3_ on anthocyanin accumulation under normal light condition (Choi et al. 2002 ; Li et al. 2020; Kuhn et al. 2021), but the crosstalk between the GA signal and light signal during sweet cherry fruit coloration remains largely unknown.

C-REPEAT BINDING FACTOR (CBF, also known as DEHYDRATION RESPONSIVE ELEMENT-BINDING PROTEIN1 [DREB1]) in Arabidopsis belongs to the DREB subfamily of the AP2/ERF (APETALA2/ETHYLENE-RESPONSIVE FACTOR) transcription factor family. Recent studies have shown that *DREB1s* respond to light. For instance, OsDREB1C in rice (*Oryza sativa*) and CBF1 in Arabidopsis enhance nitrogen use efficiency and promote hypocotyl elongation, respectively, in response to light (Dong et al. 2020; Wei et al. 2022). Additionally, DREBs regulate anthocyanin biosynthesis in Arabidopsis and in the leaves and stems of strawberry (*Fragaria × ananassa*) (Gu et al. 2015; Ren et al. 2019; Zhou et al. 2020; Luo et al. 2024). However, it is unclear whether DREBs contribute to anthocyanin accumulation in fruits in response to light and GA signaling pathways, and the regulatory mechanism of which remains poorly understood.

In this study, we identified PavDREB1E as a key component that responds to light and GA_3_ levels to positively regulate anthocyanin biosynthesis in sweet cherry fruits by directly binding to the promoters of anthocyanin biosynthesis genes. Furthermore, PavDREB1E interacts with the GA-related DELLA PavDWARF8 (PavD8) to enhance its own transcriptional activation activity, and PavHY5 strengthens this interaction. Importantly, these 3 proteins are degraded in the dark via PavCOP1-1 and PavCOP1-2. Thus, we propose a molecular model in which the PavDREB1E–PavD8–PavHY5 module integrates GA and light signals to modulate anthocyanin biosynthesis in sweet cherry fruits.

## Results

### Light promotes anthocyanin biosynthesis and reduces GA_3_ content in sweet cherry fruits

**Figure 1.**
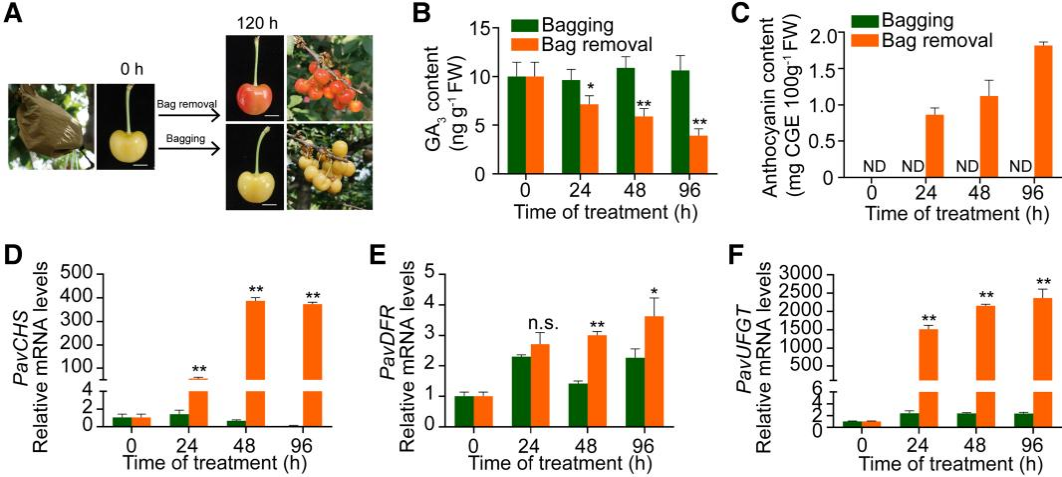
Exploration of fruit coloration in sweet cherry following light exposure. **A)** Representative photographs of sweet cherry fruits (*P. avium* cv. “Rainier”) following dark treatment only (bagging) or exposure to light after bag removal. Scale bars, 1 cm. **B** and **C)** Quantification of total GA_3_ or total anthocyanin levels in Rainier fruits maintained in the dark (bagging) or exposed to light for up to 96 h (bag removal). ND, not detected. **D** to **F)** RT-qPCR analysis of the relative transcript levels of the key anthocyanin biosynthesis-related genes *PavCHS*, *PavDFR*, and *PavUFGT*. *PavACTIN1* served as an internal control. In **B)** to **F)**, values are means ± Sd of 3 biological replicates (15 fruits per replicate). The significance of differences was determined using Student's *t*-test (*P < 0.05, **P < 0.01; n.s., not significant).

To study the effect of light on anthocyanin biosynthesis in sweet cherry fruits, we placed the fruits of the bicolored sweet cherry cultivar Rainier in black bags at 15 d after flowering (DAF). We then removed half of the bags at 45 DAF to expose the fruits to light. The bagged cherries were free of anthocyanins and had a yellow-green color, whereas fruits whose bags were removed rapidly accumulated anthocyanins within 5 d of bag removal and acquired a redder coloration (Fig. 1, A and C). RT-qPCR analysis of the expression levels of the key anthocyanin biosynthesis genes *PavCHS*, *PavDFR*, and *PavUFGT* revealed that light exposure induced the expression of these genes, corresponding to the same pattern observed for anthocyanin accumulation (Fig. 1, D to F). Notably, *PavUFGT* transcript levels increased more than 2,000-fold in response to light exposure (Fig. 1F). Furthermore, GA_3_ levels dropped significantly in fruits whose bags were removed compared to fruits that remained in bags and GA_3_ content shows high negatively correlated with anthocyanin content, suggesting that lower GA_3_ levels may contribute to light-induced anthocyanin accumulation in bicolored sweet cherry fruits (Fig. 1B; Supplementary Fig. S1).

### GA_3_ signals repress light-induced anthocyanin biosynthesis

**Figure 2.**
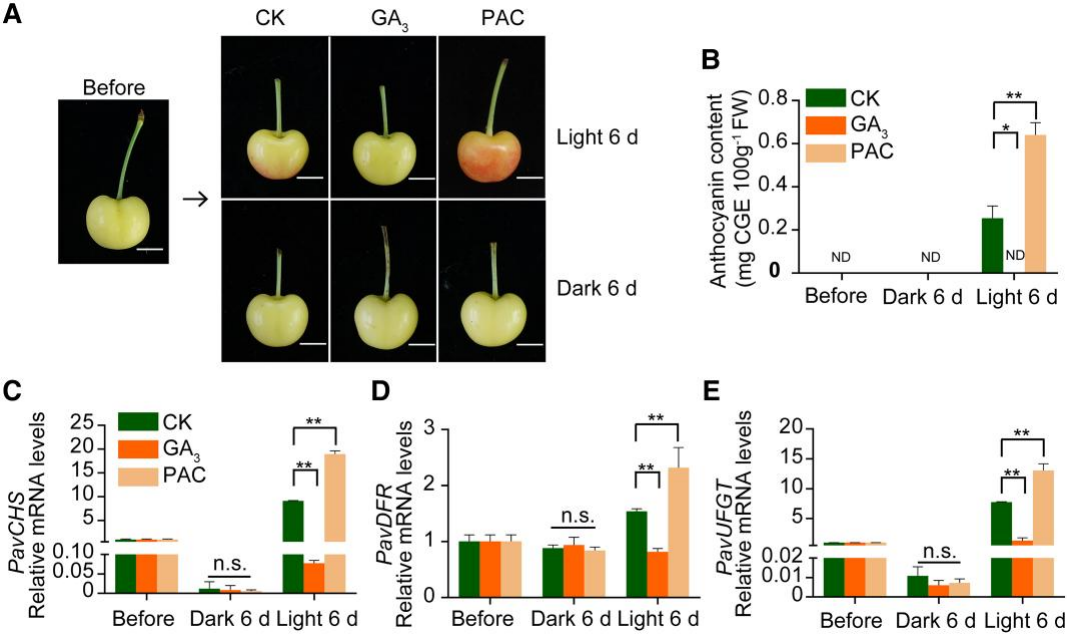
Effects of light and GA_3_ treatments on sweet cherry fruit coloration. **A)** Representative photographs of Rainier fruits subjected to light and GA_3_ treatments. Fruits were treated with H_2_O, GA_3_, or PAC in the light or dark. CK, control; GA_3_, gibberellic acid; PAC, paclobutrazol. Scale bar, 1 cm. **B)** Quantification of anthocyanin contents in sweet cherry fruits under various conditions. CGE, cyanidin-3-galactoside equivalents; FW, fresh weight; ND, not determined. **C** to **E)** Relative expression levels of the key anthocyanin biosynthesis genes *PavCHS*, *PavDFR*, and *PavUFGT* in response to various treatments, as determined by RT-qPCR. *PavACTIN1* served as an internal control. In **B)** to **E)**, values are means ± Sd of 3 biological replicates (15 fruits per replicate). The significance of differences was assessed using Student's *t*-test (*P < 0.05, **P < 0.01; n.s., not significant).

To investigate the role of GA_3_ in light-mediated anthocyanin accumulation, we harvested Rainier cherry fruits at 49 DAF and subjected them to light or dark conditions, in combination with H_2_O (as a control, CK), GA_3_, or paclobutrazol (PAC, an inhibitor of GA biosynthesis) treatment (Fig. 2A). After 6 d of treatment, we assessed the anthocyanin contents and the expression levels of anthocyanin biosynthesis genes in fruits. In the light, control fruits showed some anthocyanin accumulation, while PAC treatment resulted in an enhanced red coloration; by contrast, GA_3_ treatment suppressed fruit coloration (Fig. 2A). These coloration patterns were associated with notable differences in anthocyanin levels (Fig. 2, A and B). In the dark, however, none of the fruits exhibited any red coloration or anthocyanin accumulation (Fig. 2, A and B). *PavCHS*, *PavDFR*, and *PavUFGT* were downregulated by GA_3_ treatment and upregulated by PAC treatment compared to control fruits in the light, while no significant differences were observed in the dark (Fig. 2, C to E).

### PavDREB1E positively regulates light-induced anthocyanin biosynthesis in bicolored sweet cherry fruits

Transcription factors are critical to the regulation of anthocyanin biosynthesis, particularly in response to environmental cues (Shen et al. 2014; Tao et al. 2020; Yang et al. 2021; Xing et al. 2022; Wang et al. 2023b). We wished to identify transcription factors involved in light-induced anthocyanin accumulation. Previous studies have highlighted the roles of DREBs in light responses, such as OsDREB1C in rice, which enhances nitrogen use efficiency, and CBF1 in Arabidopsis, which promotes hypocotyl elongation in the light at ambient temperatures (Dong et al. 2020; Wei et al. 2022). Besides, DREBs regulate anthocyanin biosynthesis in Arabidopsis and in the leaves and stems of strawberry (Gu et al. 2015; Ren et al. 2019; Zhou et al. 2020; Luo et al. 2024). Additionally, transcriptome data generated by our laboratory suggested that PavDREB family genes might function in light responses (Guo et al. 2018). Accordingly, we looked for the PavDREB genes in the sweet cherry genome and identified 26 such genes (Supplementary Fig. S2, A and B).

**Figure 3.**
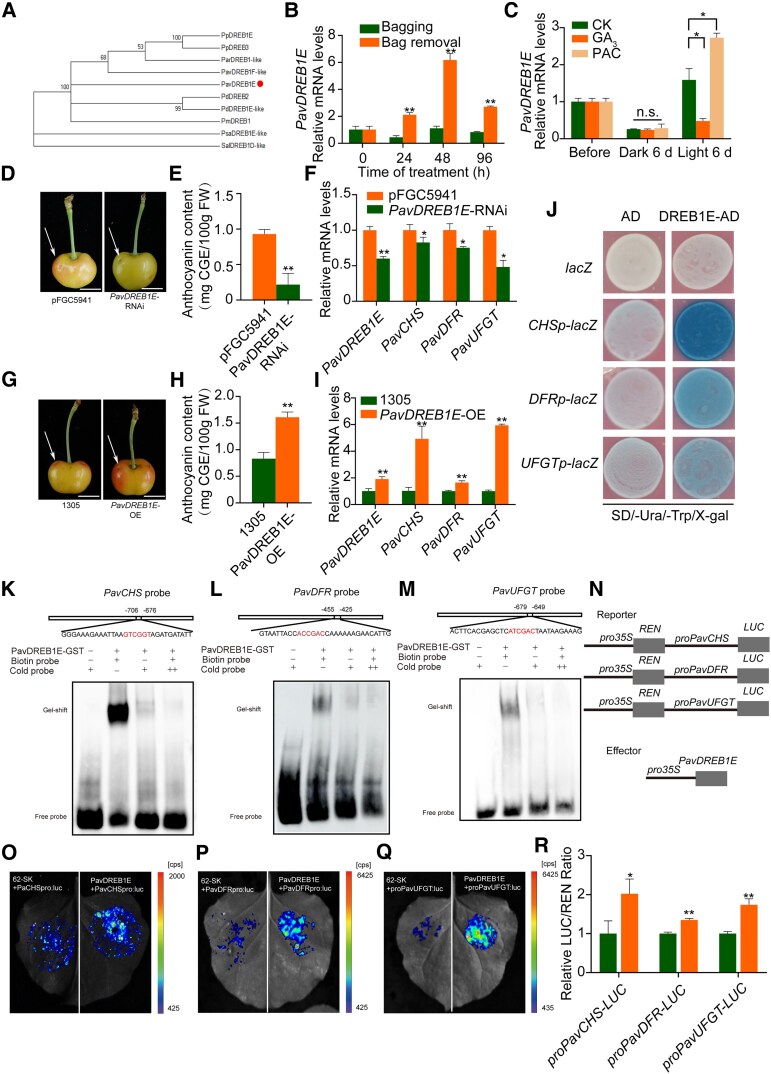
Identification and functional analysis of PavDREB1E. **A)** Phylogenetic tree of PavDREB1E. The tree was reconstructed using the maximum likelihood method in MEGA 6, with bootstrap values based on 1,000 replicates shown for each branch. The protein sequences from PavDREB1E and related proteins from peach (Pp, *Prunus persica*), apricot (Par, *Prunus armeniaca*), almond (Pd, *Prunus dulcis*), Japanese apricot (Pm, *Prunus mume*), pea (Psa, *Pisum sativum*), and scarlet sage (Sal, *Salvia splendens*) were included for comparison. **B** and **C)** Relative *PavDREB1E* transcript levels in the light or light plus GA_3_ treatments. *PavACTIN1* served as an internal control. **D)** Representative photograph showing that transient silencing of *PavDREB1E* led to lower anthocyanin biosynthesis in the peels of Rainier sweet cherry fruits. **E)** Anthocyanin contents near the infiltration sites in control and silenced fruits. **F)** Relative transcript levels of *PavDREB1E* and key anthocyanin biosynthesis genes in the peels of control and transiently silenced fruits. **G)** Representative photograph showing that transient overexpression of *PavDREB1E* led to enhanced anthocyanin biosynthesis in the peels of Rainier sweet cherry fruits. **H)** Anthocyanin contents near the infiltration sites in control and overexpressing fruits. **I)** Relative transcript levels of *PavDREB1E* and anthocyanin biosynthesis genes in the peels of transiently overexpressing fruits. *PavACTIN1* served as an internal control. **J)** Y1H assays showing the interactions of PavDREB1E with the promoters of *PavCHS*, *PavDFR*, and *PavUFGT*. **K** to **M)** EMSAs showing the binding of recombinant purified PavDREB1E-GST to probes derived from the *PavCHS*, *PavDFR*, and *PavUFGT* promoters. Unlabeled probes were used for competition, with “−” and “+” indicating absence and presence, respectively. **N)** Diagrams of *LUC* reporter constructs containing the *PavCHS*, *PavDFR*, or *PavUFGT* promoter and the *PavDREB1E* effector construct. **O** to **Q)** LUC transactivation assays showing the transactivation activity of PavDREB1E toward the *PavCHS*, *PavDFR*, and *PavUFGT* promoters. **R)** Quantification of relative LUC activity, normalized to *Renilla* luciferase (REN) activity from a *35S: REN* construct co-infiltrated with each *pro:LUC* reporter. The empty vector pGreenII 0029 62-SK was used as a control. In **B)**, **C)**, **E)**, **F)**, **H)**, **I)**, and **R)**, values are means ± Sd from 3 replicate measurements. The significance of differences was determined by Student's *t*-test (*P < 0.05, **P < 0.01; n.s., not significant).

Among these genes, *PavDREB1E* exhibited the most pronounced induction in response to light (Supplementary Fig. S3; Fig. 3, A and B). An analysis of the cis-elements presents in its promoter suggested that *PavDREB1E* might be regulated by light and GA (Supplementary Fig. S4A). Furthermore, RT-qPCR analysis revealed the upregulation of *PavDREB1E* in response to PAC treatment and its downregulation by GA_3_ treatment (Fig. 3C). To assess the subcellular localization of PavDREB1E, we generated a *35S:PavDREB1E-GFP* construct encoding a fusion of PavDREB1E and GFP. When we co-infiltrated this construct into the leaves of *Nicotiana benthamiana* plants with the nuclear marker construct *35S:NF-YA4:mCherry*, we detected green fluorescence in the nucleus and cytoplasm (Supplementary Fig. S4B). Relative *PavDREB1E* transcript levels followed the same pattern as anthocyanin levels and the transcript levels of *PavCHS*, *PavDFR*, and *PavUFGT* (Fig. 3, B and C). These results suggest that the transcription factor PavDREB1E may regulates anthocyanin biosynthesis.

To investigate whether PavDREB1E influences light-induced anthocyanin biosynthesis and fruit coloration, we performed transient overexpression and silencing of *PavDREB1E* in Rainier sweet cherry fruits. We introduced the overexpression construct pCAMBIA1305-*PavDREB1E* and the silencing construct pFGC5941-*PavDREB1E* individually into Agrobacterium (*Agrobacterium tumefaciens*) cells and infiltrated into Rainier sweet cherry fruits. RT-qPCR analysis confirmed that *PavDREB1E* transcript levels were significantly higher in *PavDREB1E*-overexpressing fruits compared to control fruits infiltrated with empty vector, while *PavDREB1E* expression was markedly lower in *PavDREB1E*-silenced fruits (Fig. 3, F and I; Supplementary Fig. S5). Silencing of *PavDREB1E* led to diminished fruit coloration compared to control fruits (Fig. 3, D and E), whereas overexpression of *PavDREB1E* enhanced fruit coloration (Fig. 3, G and H). In agreement with these observations, the expression levels and patterns of the anthocyanin biosynthesis genes *PavCHS*, *PavDFR*, and *PavUFGT* aligned with those of *PavDREB1E* and reflected anthocyanin levels (Fig. 3, F and I). These results thus suggest that PavDREB1E is involved in the light-mediated regulation of fruit coloration.

To determine whether PavDREB1E binds to the promoters of anthocyanin biosynthesis genes, we conducted a yeast 1-hybrid (Y1H) assays. Indeed, PavDREB1E directly bound to the *PavCHS*, *PavDFR*, and *PavUFGT* promoters in yeast cells (Fig. 3J). In addition to canonical DRE motifs (A/GCCGAC), which are known binding sites for DREBs, DRE-like motifs (GCCGAC and ATCGAC) have also been identified as potential binding sites for DREBs (Sakuma et al. 2002; Maruyama et al. 2004; Magome et al. 2008). We determined that the *PavCHS* and *PavDFR* promoters contain DRE motifs, while the *PavUFGT* promoter contains putative DRE-like motifs. We thus performed electrophoretic mobility shift assays (EMSAs) with recombinant purified PavDREB1E fused to glutathione S-transferase (GST) to validate the interaction between PavDREB1E and these promoters. As biotin-labeled probes, we used promoter fragments containing a DRE motif (*PavCHS*, *PavDFR*) or a DRE-like motif (*PavUFGT*). We observed a specific signal for the binding of PavDREB1E-GST to each labeled probe, which decreased in the presence of an unlabeled probe as a competitor, indicating that PavDREB1E binds directly to the *PavCHS*, *PavDFR*, and *PavUFGT* promoters in vitro (Fig. 3, K to M).

We also performed firefly luciferase (LUC) reporter assays to evaluate whether PavDREB1E activates or suppresses the transcription of anthocyanin biosynthesis genes. We placed the LUC reporter construct under the control of the *PavCHS*, *PavDFR*, or *PavUFGT* promoter, generating the reporter constructs *PavCHSpro:LUC*, *PavDFRpro:LUC*, and *PavUFGTpro:LUC*. When we co-infiltrated *PavCHSpro:LUC*, *PavDFRpro:LUC*, or *PavUFGTpro:LUC* with the *35S:PavDREB1E* effector construct into *N. benthamiana* leaves, we detected significantly greater luminescence intensity than in leaves co-infiltrated with the empty effector (Fig. 3, N to R), suggesting that PavDREB1E directly activates the transcription of *PavCHS*, *PavDFR*, and *PavUFGT*.

### PavD8 interacts with PavDREB1E and modulates its ability to activate the transcription of *PavCHS*, *PavDFR*, and *PavUFGT*

**Figure 4.**
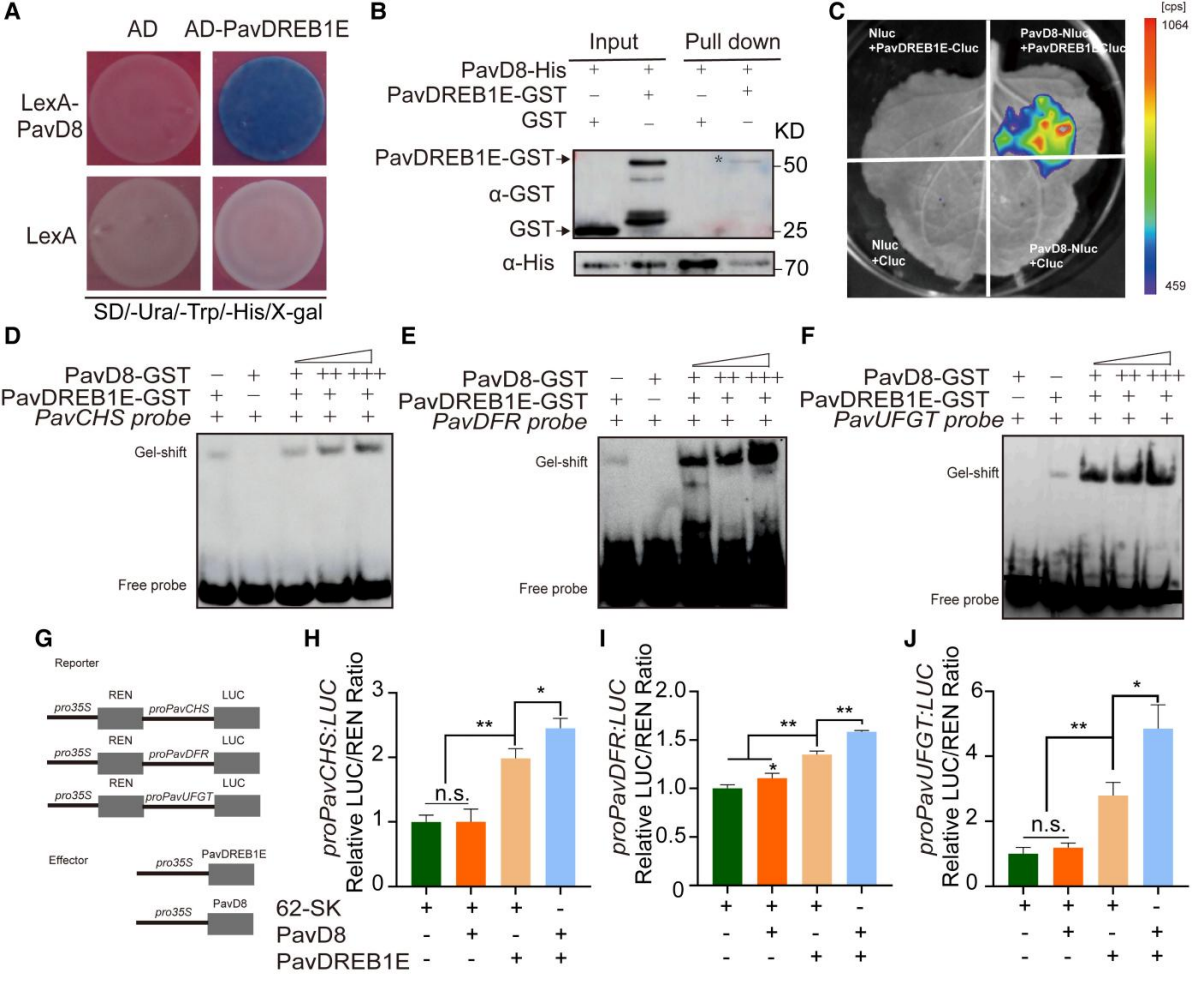
PavD8 interacts with PavDREB1E and strengthens its binding ability to its downstream target genes. **A)** Y2H assay showing the interaction between PavDREB1E and PavD8. PavD8 was fused with the LexA DNA binding domain in pEG202, and PavDREB1E was fused to the AD in pB42AD. LexA and AD served as the negative controls. **B)** His pull-down assay demonstrating the interaction between recombinant purified PavDREB1E-GST and PavD8-His. PavD8-His was incubated with GST or PavDREB1E-GST. The associated proteins were pulled down using His agarose beads and analyzed via immunoblotting with anti-GST and anti-His antibodies. **C)** LCI assay confirming the interaction between PavDREB1E and PavD8. **D** to **F)** EMSAs showing that higher concentrations of PavD8 enhance the binding of PavDREB1E to probes derived from the *PavCHS*, *PavDFR*, and *PavUFGT* promoters. The generated PavD8-GST was added to the corresponding reactions at increasing concentrations, with symbols (+, ++, +++) representing 2, 4, and 6 *μ*g of protein, respectively. **G)** Diagrams of the constructs used in the dual-LUC assays. REN, *Renilla* luciferase. **H** to **J)** Dual-LUC assay showing the synergistic effect of PavD8 on PavDREB1E-mediated transcriptional activation of the *PavCHS*, *PavDFR*, and *PavUFGT* promoters. Relative LUC activity is shown as the LUC: REN ratio, with the LUC: REN values of each *pro:LUC* co-infiltrated with the empty vector pGreenII 0029 62-SK set to 1. Values are means ± Sd from 3 biological measurements. The significance of differences was determined by Student's *t*-test (*P < 0.05, **P < 0.01; n.s., not significant).

We then examined whether DELLAs participate in light-induced PavDREB1E-mediated fruit coloration. We characterized the DELLA family in sweet cherry (Supplementary Fig. S6, A and B). We thus identified 3 DELLA family in sweet cherry, designated DWARF8 (PavD8), GA INSENSITIVE (PavGAI), and REPRESSOR OF *ga1-3* (PavRGA) based on phylogenetic analysis with related proteins from other plant species (Supplementary Fig. S6, A and B). We tested the interactions of PavDREB1E with these DELLA family members through LUC complementation imaging (LCI) assays using each protein fused to the N-terminal or C-terminal half of LUC (Fig. 4C; Supplementary Fig. S6, C and D) and determined that PavD8 and PavRGA, but not PavGAI, interact with PavDREB1E. Given the higher expression level of *PavD8* compared to *PavRGA* in fruits (Supplementary Fig. S6E), we chose PavD8 for further analysis. Subcellular localization analysis revealed that PavD8 is distributed in the nucleus and cytoplasm (Supplementary Fig. S6F). We confirmed the interaction between PavDREB1E and PavD8 through a yeast 2-hybrid (Y2H) assay and a His pull-down assay using recombinant purified PavDREB1E-GST and PavD8-His (Fig. 4, A and B).

To determine whether PavD8 affects the binding of PavDREB1E to the *PavCHS*, *PavDFR*, and *PavUFGT* promoters, we performed an additional set of EMSAs by mixing recombinant purified PavDREB1E-GST and PavD8-GST with the probes derived from the *PavCHS*, *PavDFR*, and *PavUFGT* promoters shown in Fig. 3, K to M. The addition of PavD8-GST enhanced the intensity of the shifted band corresponding to the complex between PavDREB1E and each probe (Fig. 4, D to F), indicating that PavD8 strengthens the interaction between PavDREB1E and the *PavCHS*, *PavDFR*, and *PavUFGT* promoters.

To assess the consequences of this enhanced binding on transcriptional activity, we performed a dual-LUC assay. In addition to the above reporter and effector constructs, we generated the effector construct *35S:PavD8* (Fig. 4G). Upon co-infiltrating each *LUC* reporter with *35S:PavD8* and *35S:PavDREB1E into N. benthamiana* leaves, we observed a significant increase in relative LUC activity compared to that using *35S:PavDREB1E* alone, confirming that PavD8 enhances the ability of PavDREB1E to activate transcription from the *PavCHS*, *PavDFR*, and *PavUFGT* promoters (Fig. 4, H to J). Collectively, these results suggest that PavD8 acts as a co-activator to promote the transcriptional activity of PavDREB1E on *PavCHS*, *PavDFR*, and *PavUFGT*.

### PavHY5 positively regulates light-induced anthocyanin biosynthesis by strengthening the interaction between PavD8 and PavDREB1E

**Figure 5.**
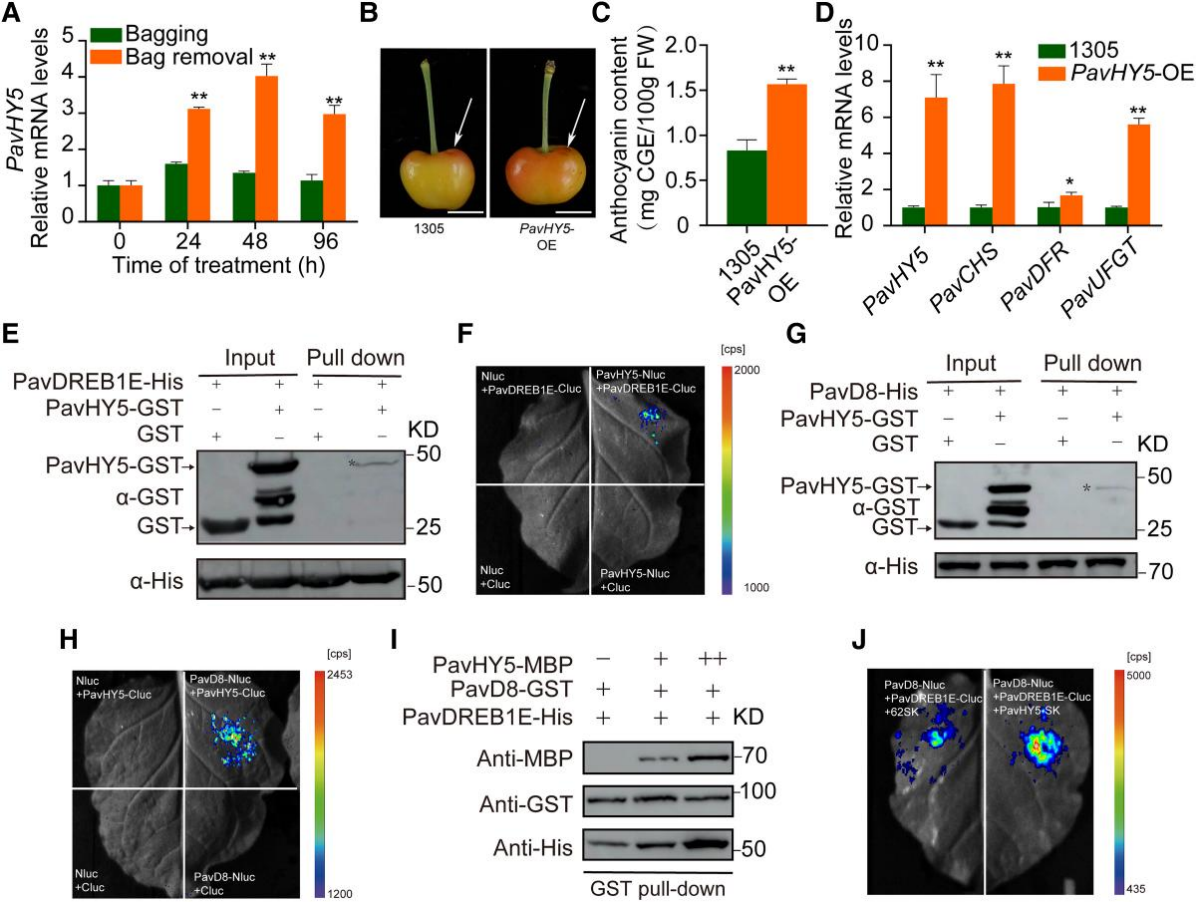
PavHY5 promotes anthocyanin accumulation by strengthening the interactions between PavD8 and PavDREB1E. **A)** Relative *PavHY5* expression levels in the light, as determined by RT-qPCR. *PavACTIN1* was used as an internal control. **B)** Transient overexpression of *PavHY5* induces anthocyanin biosynthesis in the peels of Rainier sweet cherry fruits. Scale bar, 1 cm. **C)** Anthocyanin contents near the infiltration sites in sweet cherry fruits transiently overexpressing *PavHY5*. Scale bar, 1 cm. **D)** Relative transcript levels of *PavHY5* and anthocyanin biosynthesis genes in fruits in transiently overexpressing *PavHY5*. *PavACTIN1* served as an internal control. In **A)** to **D)**, values are means ± Sd of 3 biological replicates (15 fruits per replicate). The significance of differences was determined by Student's *t*-test (*P < 0.05, **P < 0.01). **E)** His pull-down assay showing the interaction between PavHY5-GST and PavDREB1E-His. Recombinant purified PavDREB1E-His was incubated with either GST or PavHY5-GST. The interacting proteins were pulled down using His agarose beads and analyzed by immunoblotting with anti-GST or anti-His antibodies. **F)** LCI assay confirming the interaction between PavHY5 and PavDREB1E in *N. benthamiana* leaves. **G)** His pull-down assay showing the interaction between PavHY5 and PavD8. Recombinant purified PavD8-His was incubated with GST or PavHY5-GST. The interacting proteins were pulled down with His agarose beads and analyzed by immunoblotting with anti-GST or anti-His antibodies. **H)** LCI assay confirming the interaction between PavHY5 and PavD8 in *N. benthamiana* leaves. **I)** His pull-down assay showing that PavHY5 enhances the interaction between PavD8 and PavDREB1E. A mixture of PavHY5-MBP and PavD8-His was added to PavDREB1E-GST immobilized on glutathione beads. “+” and “++” indicate increasing amounts of PavHY5-MBP. **J)** LCI assay confirming that PavHY5 enhances the interaction between PavD8 and PavDREB1E in *N. benthamiana* leaves.

To more deeply explore the mechanisms underlying light-induced anthocyanin biosynthesis, we looked for HY5 in sweet cherry, as HY5 in various plant species are core regulators of light-mediated coloration (An et al. 2017; Liu et al. 2018; Fang et al. 2019; Xing et al. 2023) (Supplementary Fig. S7A). We established that *PavHY5* expression is induced by light treatment in bicolored sweet cherry fruits (Fig. 5A). PavHY5-GFP fusion protein localized to the nucleus when its encoding construct was infiltrated into *N. benthamiana* leaves (Supplementary Fig. S7B). Overexpression of *PavHY5* led to more pronounced fruit coloration and anthocyanin accumulation in fruits, with the expression of anthocyanin biosynthesis genes corresponding to the pattern of anthocyanin accumulation (Fig. 5, B to D; Supplementary Fig. S8). These findings indicate that light-induced PavHY5 positively regulates anthocyanin accumulation in bicolored sweet cherry fruits.

To elucidate the role of PavHY5 in PavDREB1E–PavD8-mediated anthocyanin accumulation, we performed His pull-down and LCI assays to assess the potential interactions between PavHY5 and PavD8 or PavDREB1E. PavHY5 interacted with PavD8 and PavDREB1E in both assays (Fig. 5, E to H). Moreover, the PavD8–PavDREB1E interaction was enhanced in the presence of PavHY5, as demonstrated by pull-down and LCI assays (Fig. 5, I and J). Collectively, these findings suggest that PavHY5 increases anthocyanin biosynthesis (at least in part) by strengthening the interaction between PavD8 and PavDREB1E. The PavDREB1E–PavD8–PavHY5 module thus appears to contribute to the light-mediated anthocyanin accumulation in sweet cherry fruits.

### Light treatment influences the stability of PavDREB1E, PavD8, and PavHY5

**Figure 6.**
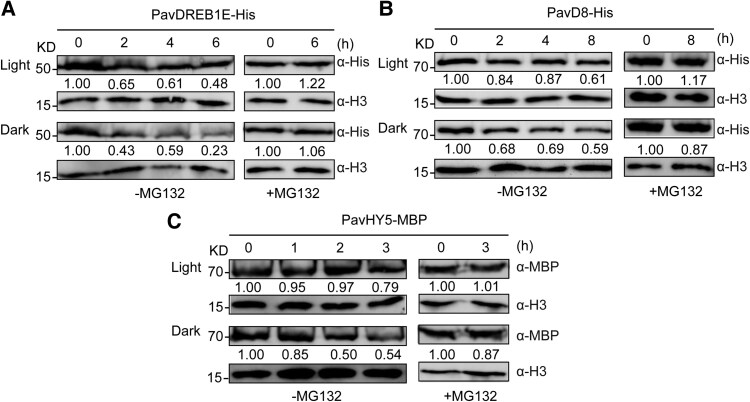
PavDREB1E-His, PavD8-His, and PavHY5-MBP are degraded more slowly by nuclear proteins extracted from sweet cherry fruits exposed to light vs. fruits maintained in the dark. **A** to **C)** Cell-free degradation assays of recombinant purified PavDREB1E-His, PavD8-His, and PavHY5-MBP. Each recombinant protein was incubated with nuclear proteins extracted from sweet cherry fruits exposed to light or maintained in the dark, together with 50 *µ*M MG132 or DMSO only (carrier control). PavDREB1E-His and PavD8-His were detected with anti-His antibodies, while PavHY5-MBP was detected with anti-MBP antibodies. Anti-histone H3 was used as a loading control. The band intensity was measured using ImageJ software.

To investigate the stability of each component of the PavDREB1E–PavD8–PavHY5 module upon light exposure, we extracted nuclear proteins from sweet cherry fruits subjected to light or dark treatment, as transcription factors primarily function within the nucleus. We then performed cell-free degradation assays using these nuclear extracts, to which we added recombinant purified PavDREB1E-His, PavD8-His, or PavHY5-MBP. PavDREB1E-His, PavD8-His, and PavHY5-MBP degraded more rapidly when incubated with nuclear extracts derived from dark vs. light-treated sweet cherry fruits, and this degradation was inhibited by treatment with the 26S proteasome inhibitor MG132 (Fig. 6, A to C).

### PavCOP1-1 and PavCOP1-2 ubiquitinate the PavDREB1E–PavD8–PavHY5 module in sweet cherry fruits

Based on the above findings, we focused on COP1, the hub protein of the light signaling cascade. This E3 ubiquitin ligase directly interacts with and is responsible for the ubiquitination of transcription factors with positive roles in various plant species, such as HY5, MYBs, and PpbHLH64, which promote anthocyanin biosynthesis, leading to their proteasomal degradation (An et al. 2019; Bhatnagar et al. 2020; Tao et al. 2020). Importantly, COP1-mediated protein degradation is modulated by its dark-induced nuclear accumulation (von Arnim and Deng 1994). We identified 2 COP1 genes in sweet cherry, designated PavCOP1-1 and PavCOP1-2 (Supplementary Fig. S9, A and B).

**Figure 7.**
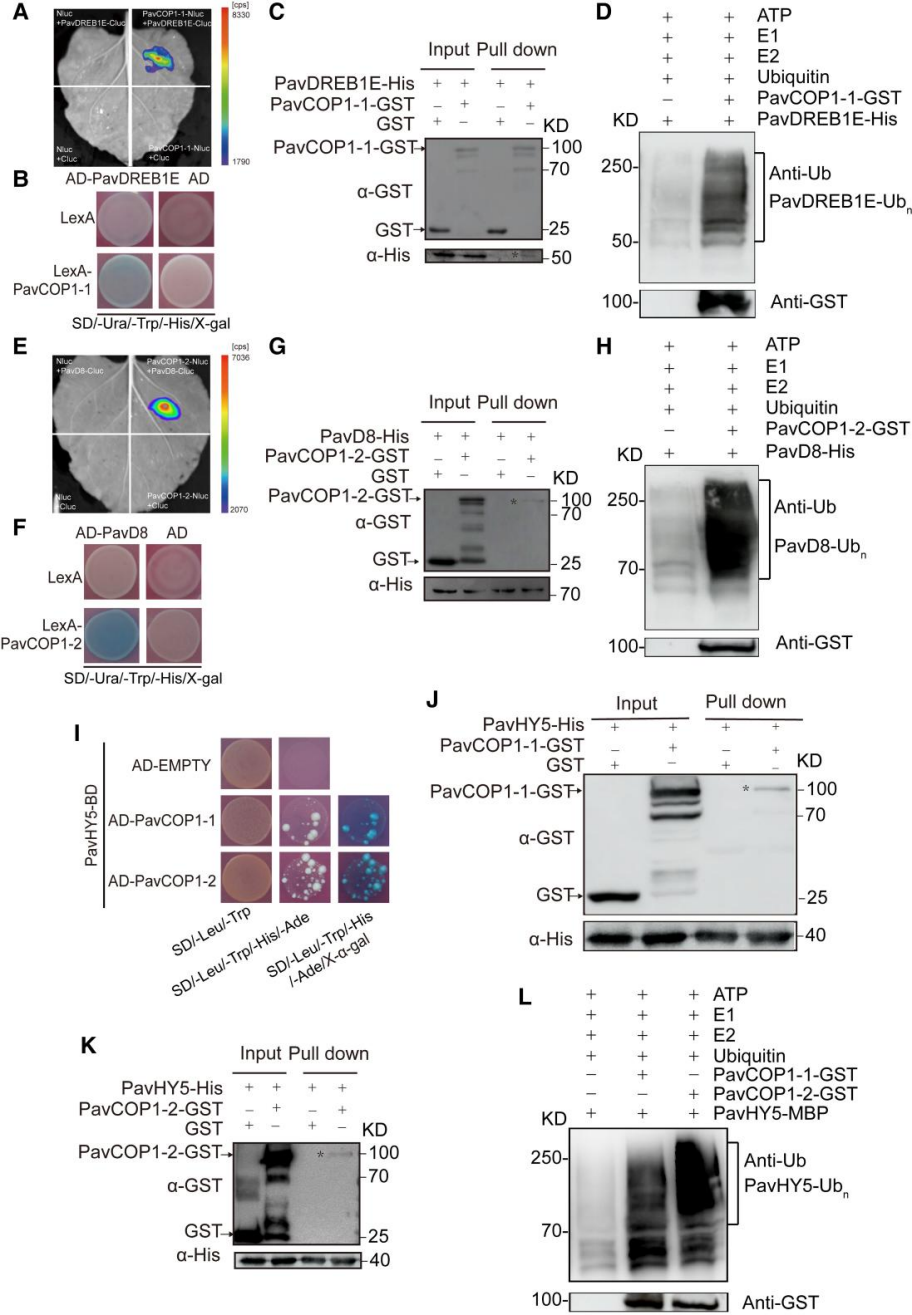
PavCOP1-1 and PavCOP1-2 ubiquitinate PavDREB1E, PavD8, and PavHY5. **A** to **C)** PavDREB1E and PavCOP1-1 interact, as determined by LCI assay, Y2H assay, and GST pull-down assay. **D)** PavCOP1-1 ubiquitinates PavDREB1E in vitro. Recombinant purified PavDREB1E-His was used to detect potential E3 ubiquitin ligase activity in the presence of ATP, ubiquitin (Ub), an E1 Ub-activating enzyme, an E2 Ub-conjugating enzyme, and recombinant PavCOP1-1-GST. Immunoblot analysis with an anti-Ub antibody was performed to detect PavDREB1E ubiquitination. **E** to **G)** PavD8 and PavCOP1-2 interact, as determined by LCI assay, Y2H assay, and His pull-down assay. **H)** PavCOP1-2 ubiquitinates PavD8 in vitro. Recombinant purified PavD8-His was used to detect potential E3 Ub ligase activity in the presence of ATP, Ub, E1, E2, and recombinant PavCOP1-2-GST. Immunoblot analysis with an anti-Ub antibody was performed to detect PavD8 ubiquitination. **I** to **K)** PavHY5 and PavCOP1-1 or PavCOP1-2 interact, as determined by a Y2H assay and His pull-down assays. **L)** PavCOP1-1 and PavCOP1-2 ubiquitinate PavHY5 in vitro. Recombinant purified PavHY5-MBP was used to detect potential E3 Ub ligase activity in the presence of ATP, Ub, E1, E2, and recombinant PavCOP1-1-GST or PavCOP1-2-GST. Immunoblot analysis with an anti-Ub antibody was performed to detect PavHY5 ubiquitination.

To investigate whether PavCOP1-1 and/or PavCOP1-2 affect the abundance of each component of the PavDREB1E–PavD8–PavHY5 module through direct interactions, we performed LCI assays in *N. benthamiana* leaves. PavDREB1E interacted with PavCOP1-1 but not PavCOP1-2, whereas PavD8 interacted with PavCOP1-2 but not PavCOP1-1 (Fig. 7, A and E; Supplementary Fig. S9, C and D). Y2H assays and pull-down assays confirmed the interactions between PavDREB1E and PavCOP1-1 and between PavD8 and PavCOP1-2 (Fig. 7, B, C, F, and G). Additionally, both PavCOP1-1 and PavCOP1-2 interacted with PavHY5 in Y2H and pull-down assays (Fig. 7, I to K). In in vitro ubiquitination assays, recombinant purified PavCOP1-1-GST ubiquitinated PavDREB1E-His when co-incubated with ATP, an E1 ubiquitin-activating enzyme, an E2 ubiquitin-conjugating enzyme, and ubiquitin (Fig. 7D), while PavCOP1-2-GST facilitated the ubiquitination of PavD8-His in a similar assay (Fig. 7H). Moreover, the addition of either PavCOP1-1 or PavCOP1-2 led to the ubiquitination of PavHY5-MBP in vitro, as evidenced by immunoblot analysis with an anti-Ub antibody (Fig. 7L). Collectively, these results suggest that PavCOP1-1 and PavCOP1-2 inhibit fruit coloration by mediating the degradation of PavDREB1E, PavD8, and PavHY5 in sweet cherry fruits.

## Discussion

Several studies have highlighted the pivotal roles of light in modulating anthocyanin production and fruit pigmentation in various plant species (Tao et al. 2020; Hu et al. 2021; Yang et al. 2021; Liu et al. 2024). Light-induced coloration is governed by various transcription factors, such as HY5 and members of the BBX, MYB, and bHLH families (Tao et al. 2020; Yang et al. 2021 ; Xing et al. 2022; Wang et al. 2023b; Liu et al. 2024). However, other potential light-responsive transcription factors in coloration have not been fully characterized. In this study, we demonstrated that the transcription factor PavDREB1E is responsive transcriptionally to light and positively regulates anthocyanin biosynthesis in bicolored sweet cherry fruits by binding directly to the promoters of key anthocyanin biosynthesis genes *PavCHS*, *PavDFR*, and *PavUFGT* (Fig. 3).

Light regulates anthocyanin biosynthesis together with various phytohormones, including jasmonate, ethylene, brassinosteroids, and abscisic acid (Liu et al. 2015; An et al. 2017; Samkumar et al. 2021; Wang et al. 2023a, 2023b; Li et al. 2024; Zhang et al. 2024). Furthermore, GA and light signals have opposite effects on seedling photomorphogenesis. For example, lower GA levels induce photomorphogenesis in pea (*Pisum sativum*) seedlings maintained in the dark, and DELLAs inhibit hypocotyl growth in Arabidopsis in the light (Alabadí et al. 2004, 2008; Xu et al. 2014). Nevertheless, the molecular mechanisms by which GA and light signaling interacting to regulate fruit anthocyanin biosynthesis have been largely unknown.

Here, we demonstrated that light exposure leads to lower GA_3_ contents in bicolored sweet cherry fruits (Fig. 1B), thereby stabilizing DELLAs, and GA_3_ treatment inhibits light-induced anthocyanin accumulation (Fig. 2, A and B). Since GA_3_ was detected in sweet cherry fruits (Fig. 1B) and GA_3_, as the biologically active form of GAs, has been extensively documented to regulate anthocyanin accumulation in fruit species, including apple (An et al. 2024a), litchi (Qu et al. 2022), and sweet cherry (Choi et al. 2002; Li et al. 2020; Kuhn et al. 2021), we used GA_3_ as the treatment here. Additionally, DELLA protein PavD8 strengthens the direct binding of PavDREB1E, which is encoded by a light-responsive gene that is upregulated by light but downregulated by GA_3_ treatment (Fig. 3, B and C), to the promoters of *PavCHS*, *PavDFR*, and *PavUFGT* (Fig. 4, D to J). Our results suggest 2 mechanisms by which light promotes anthocyanin accumulation: (i) light induces the transcription of *PavDREB1E*, thereby promoting anthocyanin biosynthesis, and (ii) light reduces GA_3_ levels, leading to DELLA stabilization, which enhances the binding ability of PavDREB1E to its downstream targets, thus promoting fruits coloration. However, exogenous GA_3_ treatment inhibits this process by reducing *PavDREB1E* transcription and destabilizing DELLA proteins, ultimately suppressing anthocyanin accumulation. In the dark, transcription of *PavDREB1E* and *PavHY5* is significantly reduced (Figs. 3B and 5A). The stability of PavDREB1E, PavD8, and PavHY5 proteins is weakened through ubiquitination mediated by PavCOP1-1 and PavCOP1-2 (Figs. 6 and 7). Consequently, even PAC treatment fails to promote fruit coloration due to the dual suppression of transcriptional activity and protein stability.

ABA plays a crucial role in sweet cherry fruit coloration (Shen et al. 2014, 2017; Wang et al. 2023b). In particular, we found that light induces ABA accumulation and anthocyanin biosynthesis in bicolored sweet cherry fruit (Wang et al. 2023b). To explore whether ABA is involved in the PavDREB1E-mediated regulation of anthocyanin biosynthesis, we first examined *PavDREB1E* transcriptional responses to ABA treatment under light. The results showed that ABA increased anthocyanin levels and *PavDREB1E* transcription (Supplementary Fig. S10, A to C). Furthermore, to determine whether PavDREB1E binds to the core ABA biosynthesis gene *PavNCED1* (Wang et al. 2023b), we performed Y1H assays. These assays revealed that PavDREB1E does not bind to the *PavNCED1* promoter (Supplementary Fig. S10D). These findings suggested that ABA is involved in the PavDREB1E-mediated regulation of anthocyanin biosynthesis, but PavDREB1E does not bind to the core ABA biosynthesis gene *PavNCED1*.

HY5, a hub protein in light signaling pathways, plays important roles in light and phytohormone signaling pathways by interacting with other proteins and modulating downstream pathways (An et al. 2017; Liu et al. 2018; Fang et al. 2019; Xing et al. 2023). Some studies have demonstrated that HY5 participates in GA biosynthesis (Weller et al. 2009; Gangappa and Botto 2016; Burman et al. 2018; Fu et al. 2024). However, the involvement of HY5 in GA-light signal crosstalk during anthocyanin accumulation remains poorly understood. In this study, we showed that PavHY5 enhances anthocyanin accumulation by promoting the formation of the PavDREB1E–PavD8 module through direct interactions with each protein, thereby acting as a cofactor in the GA–light-mediated modulation of anthocyanin biosynthesis (Fig. 5, B to J).

The E3 ubiquitin ligase COP1 participates in a wide range of cellular and physiological responses in plants. COP1 targets multiple proteins for ubiquitination and subsequent degradation through interactions with SUPPRESSOR OF PHYA-105 (SPA) proteins. COP1 localizes to the nucleus in the dark and migrates to the cytosol in the light (von Arnim and Deng 1994). Light-activated photoreceptors, such as red/far-red light phytochromes (PHYs), blue light receptors (cryptochromes 1 and 2 [CRY1/CRY2]), and the ultraviolet B (UV-B) light receptor (UV RESISTANCE LOCUS 8 [UVR8]), inhibit the interaction between COP1 and its targets through higher-affinity cooperative binding to COP1 (Wang et al. 2001, 2022; Kang et al. 2009; Rizzini et al. 2011; Zuo et al. 2011; Cloix et al. 2012; Lu et al. 2015; Yin et al. 2015; Lau et al. 2019; Ponnu et al. 2019; Trimborn et al. 2025). Thus, it controls the accumulation of its target proteins in the light, such as MYB (Li et al. 2012), HY5 (Osterlund et al. 2000), HYH (Holm et al. 2002), BBX proteins (Xu et al. 2016; Lin et al. 2018; Song et al. 2020), and bHLH proteins (Tao et al. 2020). COP1 also contributes to GA_3_ signaling by interacting with DELLAs (Blanco-Touriñán et al. 2020; Lee et al. 2022). Additionally, GA stabilizes COP1, which in turn leads to the destabilization of REPRESSOR OF GA-LIKE2 (RGL2) (Lee et al. 2022). However, whether and how COP1 regulates light-regulated anthocyanin biosynthesis via GA signaling has been unclear. Here, we identified 2 COP1 genes in sweet cherry fruits, PavCOP1-1 and PavCOP1-2 (Supplementary Fig. S9, A and B). Both genes interacted with PavHY5, while PavCOP1-1 and PavCOP1-2 interacted with PavD8 and PavDREB1E, respectively, leading to their ubiquitination and proteasomal degradation (Fig. 7). These findings indicate that PavCOP1-1 and PavCOP1-2 are involved in regulating the PavDREB1E–PavD8–PavHY5 module, which integrates light and GA signals to regulate anthocyanin biosynthesis in sweet cherry fruits.

**Figure 8.**
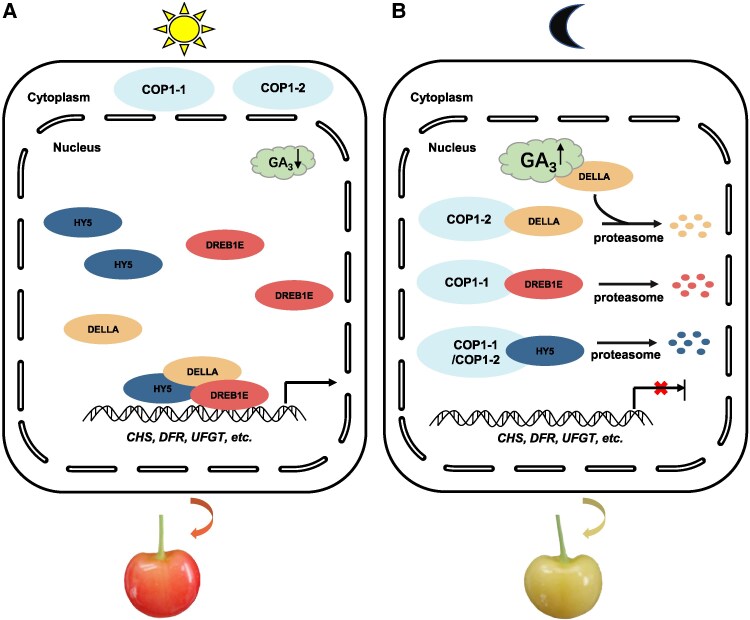
A proposed model of the PavDREB1E–PavD8–PavHY5 module in regulating light-induced fruit coloration. **A)** Light-exposed condition: Under light exposure, transcript levels of *PavDREB1E* and *PavHY5* increase, accompanied by a decrease in GA_3_ concentration. Reduced GA_3_ stabilizes the DELLA protein PavD8, which facilitates formation of the PavDREB1E–PavD8–PavHY5 transcriptional module. This module activates key anthocyanin biosynthesis genes (e.g. *CHS*, *DFR*, *UFGT*), driving fruit pigmentation. **B)** Dark condition: In continuous darkness, *PavDREB1E* and *PavHY5* transcripts decline, while GA_3_ levels rise. Elevated GA_3_ triggers destabilization of PavD8, impairing assembly of the transcriptional module. Concurrently, PavCOP1-1 and PavCOP1-2 promote ubiquitination and proteasomal degradation of module components (PavDREB1E, PavD8, and PavHY5). The resulting depletion of these factors suppresses anthocyanin gene expression, leading to reduced anthocyanin accumulation and attenuated fruit coloration in the dark.

In conclusion, our study revealed that light led to higher transcript levels of *PavDREB1E* and *PavHY5*. PavDREB1E directly binds to the promoters of key anthocyanin biosynthesis genes, promoting anthocyanin accumulation. In addition, light exposure results in lower GA_3_ levels in Rainer sweet cherry fruits, leading to the stabilization of PavD8, which enhances the promoter-binding activity of PavDREB1E through a direct protein–protein interaction. Additionally, PavHY5 strengthens the interaction between PavD8 and PavDREB1E, further promoting anthocyanin biosynthesis (Fig. 8A). In the dark, the transcript levels of both *PavDREB1E* and *PavHY5* are lower, and elevated GA_3_ levels destabilize PavD8. At the same time, PavCOP1-1 and PavCOP1-2 likely migrate from the cytosol to the nucleus in the dark, leading to the ubiquitination and proteasomal degradation of PavDREB1E, PavD8, and PavHY5 (Fig. 8B). Collectively, these findings suggest that the PavDREB1E–PavD8–PavHY5 module integrates light and GA signaling pathways to regulate anthocyanin biosynthesis in sweet cherry fruits.

## Materials and methods

### Plant materials and treatments

Bicolored sweet cherry (*P. avium* cv. Rainier) fruits were obtained from the Beijing Institute of Forestry and Pomology. For light and dark treatments, the fruits were bagged at 15 DAF. We then removed half of the bags at 45 DAF to expose the fruits to light as light treatment, and the rest half remained bagged as dark treatment. Samples were collected at 0, 24, 48, and 96 h after treatment, using 3 biological replicates (15 fruits per replicate) for each designated time point. The sampled fruits were immediately frozen in liquid nitrogen and stored at −80 °C.

For the light and GA_3_ treatments, the fruits were harvested at 49 DAF and put in the dark for 1 d to eliminate the difference between individuals and treatment with GA_3_ or PAC under light or dark conditions for 6 d. The light treatment was conducted at 25 °C under 16 h: 8 h, light: dark photoperiod (100 to 150 *μ*mol m^−2^ s^−1^), whereas the dark treatment was conducted at 25 °C under conditions of continuous darkness.

For the ABA treatments, the fruits were harvested at 49 DAF and put in the dark for 1 d to eliminate the difference between individuals and treatment with ABA or H_2_O under light conditions for 6 d. The light treatment was conducted at 25 °C under 16 h: 8 h, light: dark photoperiod (100 to 150 *μ*mol m^−2^ s^−1^).

### Anthocyanin and GA_3_ content measurements

The extraction and measurement of the total anthocyanin content were performed following the method described as previously reported (Wang et al. 2023b). The extraction and determination of endogenous GA_3_ levels were performed as previously reported (Yue et al. 2023), with GA_3_ using as the internal standard for the quantification of GA levels.

### Total RNA extraction and reverse transcription quantitative PCR

Total RNA from samples was extracted using TRIzol Reagent (CWBIO, Beijing, China). Complementary DNA (cDNA) was synthesized using the HiScript II Q RT SuperMix for qPCR (Vazyme, Nanjing, China) according to the manufacturer's protocol. RT-qPCR was performed in a 10 *μ*L reaction system using 2×HQ SYBR qPCR Mix (Zoman Biotechnology, Beijing, China) with the Rotor-Gene Real-Time PCR System. *PavACTIN1* was chosen for internal control (Wang et al. 2023b). Relative expression levels were measured by using the 2^−ΔΔCt^ method (Livak and Schmittgen 2001). All the primers used are listed in Supplementary Table S1.

### Identification and analysis of DREB family members

The 56 *DREB* genes in Arabidopsis thaliana were used as query sequences for *PavDREB* in the sweet cherry fruit genome database (http://cherry.kazusa.or.jp/). The phylogenetic tree was constructed with the neighbor-joining algorithm using MEGA 6.0 software, with bootstrap values based on 1,000 replicates shown for each branch (Tamura et al. 2013).

### Transient transformation analysis in sweet cherry

To generate pCambia1305-*PavDREB1E* and pCambia1305-*PavHY5* overexpression vectors, the CDSs of *PavDREB1E* and *PavHY5* were cloned and inserted into the *Bgl*II site of the pCambia1305 vector. Empty pCambia1305 vector was used as a negative control. The construction of the *PavDREB1E*-RNAi plasmid was performed following the protocol described as previously reported (Wang et al. 2023b). The recombinant plasmids and empty vectors were transfected into *A. tumefaciens* strain EHA105, then injected into the Rainer fruit on trees using 100 *μ*L syringes at 20 DAF (Shen et al. 2014). After photographing, the injection area of the peels was collected. All the primers used in are listed in Supplementary Table S1.

### Protein subcellular localization

The nuclear marker NF-YA4-mCherry (Yue et al. 2023) was co-overexpressed with PavDREB1E-GFP, PavD8-GFP, PavHY5-GFP or GFP into *N. benthamiana* leaves. The fluorescence signals were detected by using an Olympus FluoView 3000 confocal microscope described as previously reported (Feng et al. 2023).

### Y1H assay

The full-length CDSs of *PavDREB1E* was ligated into the pB42AD vector. The 2,000-bp promoters of *PavUFGT*, *PavCHS*, and *PavDFR* were amplified from Rainier genomic DNA and inserted into the pLacZi2μ vector. The Y1H assays were performed as described as previously reported (Zhai et al. 2022). All the primers used are listed in Supplementary Table S1.

### EMSA

The CDSs of *PavDREB1E*, *PavD8*, and *PavHY5* were fused to the pGEX-4T and pMAL-C2X vectors for expression in *Escherichia coli* Rosetta (DE3) cells to generate PavDREB1E-GST, PavD8-GST, and PavHY5-MBP fusion proteins, respectively. EMSAs were performed using a chemiluminescent EMSA Kit (Beyotime, Shanghai, China) in accordance with the manufacturer's instructions (Zhai et al. 2022). All the primers used are listed in Supplementary Table S1.

For the analysis of PavD8's effect on the binding of PavDREB1E to the promoters of *PavCHS*, *PavDFR*, and *PavUFGT*, the amount of PavDREB1E-GST protein used in each reaction was 2 *µ*g. And the generated PavD8-GST was added to the corresponding reactions at increasing concentrations (0, 2, 4, and 6 *μ*g).

### Dual-LUC assay

The 2,000-bp promoters of *PavUFGT*, *PavCHS*, and *PavDFR* were cloned and ligated into pGreenII 0800-LUC reporter vectors. The CDSs of *PavDREB1E* and *PavHY5* were inserted into the pGreenII 0029 62-SK effector vectors. Each construct was separately transformed into *A. tumefaciens* strain GV3101 (Psoup) and subsequently co-infiltrated into *N. benthamiana* leaves. The agroinfiltrated *N. benthamiana* were incubated for 2 d. The infiltrated leaves were imaged using Multi Tanon-5200 Chemiluminescent Imaging System (Tanon Science & Technology) after being sprayed with 25 mm luciferin and placed in the dark for 10 min. In addition, the injected *N. benthamiana* leaves were collected, and the fluorescence activity was detected according to the manufacturer's instructions of the fluorescence detection kit (Promega, Madison, WI, USA). All the primers used are listed in Supplementary Table S1.

### Nuclear-cytoplasmic fractionation assay

Nuclear fractionation was conducted as described as previously reported (Peng et al. 2022), using sweet cherry fruits subjected to light and dark treatment. A portion of the cytoplasmic and nuclear fractions was resuspended in 3× SDS loading buffer and boiled at 98 °C for 10 min to prepare samples for immunoblot analysis. Histone H3 proteins were detected using an anti-H3 antibody (Cell Signaling, D2B12) as a nuclear marker.

### Protein–protein interaction experiments

For in vitro pull-down assays, the experiments were performed as described previously with minor modifications (Xu et al. 2021). PavDREB1E-His, PavDREB1E-GST, PavD8-His, PavCOP1-1-GST, PavCOP1-2-GST, and PavHY5-MBP were prepared. Next, the PavDREB1E-GST, PavCOP1-1-GST, PavCOP1-2-GST, and GST protein were individually enriched with Glutathione Agarose (Thermo Scientific) and PavDREB1E-His enriched with Ni Sepharose (GE Healthcare Bio-Sciences AB). After washing the beads 3 times, the immobilized proteins were incubated with other His-, GST-, or MBP-tagged proteins. The bound proteins were subsequently eluted from the beads and subjected to immunoblot analysis using the appropriate antibodies: anti-GST (CWBIO, Beijing, Chin), anti-His (CWBIO, Beijing, China), and anti-MBP (Proteintech, Rosemont, IL, USA).

For LCI assays, the CDSs of PavDREB1E, PavD8, PavCOP1-1, PavCOP1-2, and PavHY5 were individually cloned into the JW-771-nLUC and JW-772-cLUC vectors. The constructs were introduced into *A. tumefaciens* strain GV3101 and expressed in *N. benthamiana* leaves. Chemiluminescence signals produced by LUC detected using NightSHADE LB 985 Plant Imaging System (Berthold) and Multi Tanon-5200 Chemiluminescent Imaging System (Tanon Science & Technology).

For Y2H assays, the CDSs of PavDREB1E, PavD8, PavCOP1-1 and PavCOP1-2 were individually cloned into the pEG202 (pLexA) and pB42AD vectors. Different combinations fusion plasmids and the *p8op:LacZ* reporter vector were co-transformed into yeast strain EGY48 and grown on proper drop-out plates containing 5-bromo-4-chloro-3-indolyl-β-D-galactopyranoside (X-gal) to observe the color change. The CDSs of PavCOP1-1 and PavCOP1-2 were individually cloned into the pGADT7, and the CDS of PavHY5 was cloned into the pGBKT7. Different combinations of fusion plasmids were co-transformed into the yeast strain AH109 and grown on selective medium (SD-Leu/-Trp) for screening positive clones. The positive clones were spotted onto plates of SD-Leu-Trp-His-Ade for growth, and 5-bromo-4-chloro-3-indoxyl-α-D-galactopyranoside (X-α-gal) was poured onto the plate to observe the color change. All the primers used are listed in Supplementary Table S1.

### Cell-free degradation assay

PavDREB1E-His, PavD8-His, and PavHY5-MBP fusion proteins were prepared for the cell-free degradation assays. Sweet cherry fruits in the light and dark treatment were used for nuclear/cytoplasmic fractionation assays. The fusion proteins were mixed with nuclear protein extracts and incubated at 37 °C for the specified time. Degradation was analyzed by immunoblotting using anti-His and anti-MBP antibodies. Anti-H3 served as a nuclear protein marker.

### In vitro ubiquitination assay

PavDREB1E-His, PavD8-His, PavHY5-MBP, PavCOP1-1-GST, and PavCOP1-2 GST fusion proteins were prepared for the in vitro protein degradation assays. In vitro ubiquitination assays were performed using the Recombinant Human Ubiquitin Ligase E3 (YEASEN, Shanghai, China) kit, following the manufacturer's instruction. Briefly, the reactions were prepared by combining recombinant UBE1 (E1; 2 *μ*L), UBE2D2 (E2; 2 *μ*L), PavCOP1-1-GST or PavCOP1-2-GST (E3; previously incubated with 20 *μ*M zinc acetate), ubiquitin (4 *μ*L), and purified PavDREB1E-His, PavD8-His, and PavHY5-MBP. The reactions were carried out in a buffer containing 1 mm ATP, 60 mm DTT, 500 mm Tris, and 100 mm MgCl₂ at 37 °C for 2 h. Ubiquitinated PavDREB1E-His, PavD8-His, and PavHY5-MBP were detected with the anti-ubiquitin antibodies (YEASEN, Shanghai, China).

### Statistical analyses

All data are analyzed using Student's *t*-test with GraphPad Prism version 8.0.2 (GraphPad Software, LLC, USA). Results are shown as mean values ± standard deviation (Sd). *P* < 0.05 was considered to be statistically significant.

### Accession numbers

Sequence data can be found in the NCBI website (https://www.ncbi.nlm.nih.gov/) under accession numbers PavDREB1E (LOC110747921), PavD8 (LOC110759924), PavRGA (LOC110751454), PavGAI (LOC110755123), PavCOP1-1 (LOC110764510), PavCOP1-2 (LOC110748438), PavCHS (JF748833), PavDFR (JF740093), and PavUFGT (JF740090).

## Supplementary Material

kiaf616_Supplementary_Data

## Data Availability

The data underlying this article will be shared on reasonable request to the corresponding author.

## Supplementary data

The following materials are available in the online version of this article.

**Supplementary Figure S1.** GA_3_ content is negatively correlated with anthocyanin content.

**Supplementary Figure S2.** Phylogenetic analysis and sequence alignment of the AP2 domains of DREB proteins from sweet cherry.

**Supplementary Figure S3.**  *PavDREB* transcript levels in sweet cherry fruits in response to light exposure.

**Supplementary Figure S4.** Motif analysis of the *PavDREB1E* promoter and subcellular localization of PavDREB1E.

**Supplementary Figure S5.** Confirmation of the transformation of bicolored sweet cherry fruits.

**Supplementary Figure S6.** Identification and analysis of DELLAs from sweet cherry.

**Supplementary Figure S7.** Phylogenetic analysis and subcellular localization of PavHY5.

**Supplementary Figure S8.** Validation of *PavHY5*-OE in bicolored sweet.

**Supplementary Figure S9.** Analysis of PavCOP1-1 and PavCOP1-2.

**Supplementary Figure S10.** Effects of ABA treatment on sweet cherry fruit coloration and *PavDREB1E*.

**Supplementary Table S1.** All primers used in this study.

## Dive Curated Terms

The following phenotypic, genotypic, and functional terms are of significance to the work described in this paper:

CBF1 Gramene: AT4G25490
CBF1 Araport: AT4G25490

DFR Gramene: AT5G42800
DFR Araport: AT5G42800

RGL2 Gramene: AT3G03450
RGL2 Araport: AT3G03450

ATP CHEBI: CHEBI:15422

BBX Gramene: B-box protein
BBX Araport: B-box protein

GFP Gramene: green fluorescent protein
GFP Araport: green fluorescent protein

UDP Gramene: Uridine diphosphate
UDP Araport: Uridine diphosphate

PavDREB1E Gramene: LOC110747921
PavDREB1E Araport: LOC110747921

PavCOP1-1 Gramene: LOC110764510
PavCOP1-1 Araport: LOC110764510

PavD8 Gramene: LOC110759924
PavD8 Araport: LOC110759924

PavRGA Gramene: LOC110751454
PavRGA Araport: LOC110751454

PavGAI Gramene: LOC110755123
PavGAI Araport: LOC110755123

PavCOP1-2 Gramene: LOC110748438
PavCOP1-2 Araport: LOC110748438

PavCHS Gramene: (JF748833
PavCHS Araport: (JF748833

PavDFR Gramene: JF740093
PavDFR Araport: JF740093

PavUFGT Gramene: JF740090
PavUFGT Araport: JF740090
