# Supplementary material for: The PavDREB1E–PavD8–PavHY5 module integrates light and gibberellin signals to regulate sweet cherry anthocyanin biosynthesis
Source: Plant Physiol. 2025 Nov 26;199(4):kiaf616. doi: 10.1093/plphys/kiaf616 (PMC12700780; doi:10.1093/plphys/kiaf616)
Supplement: kiaf616_Supplementary_Data [file kiaf616_supplementary_data.zip › Supplementary Data.pdf]

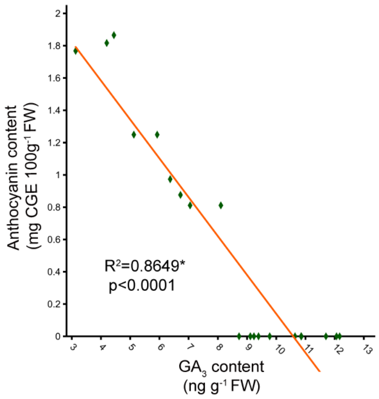

Supplementary Figure S1. GA_3 content is negatively correlated with anthocyanin content. Asterisk indicates a significant difference, as determined by Student's *t*-test; *$P < 0.05$.

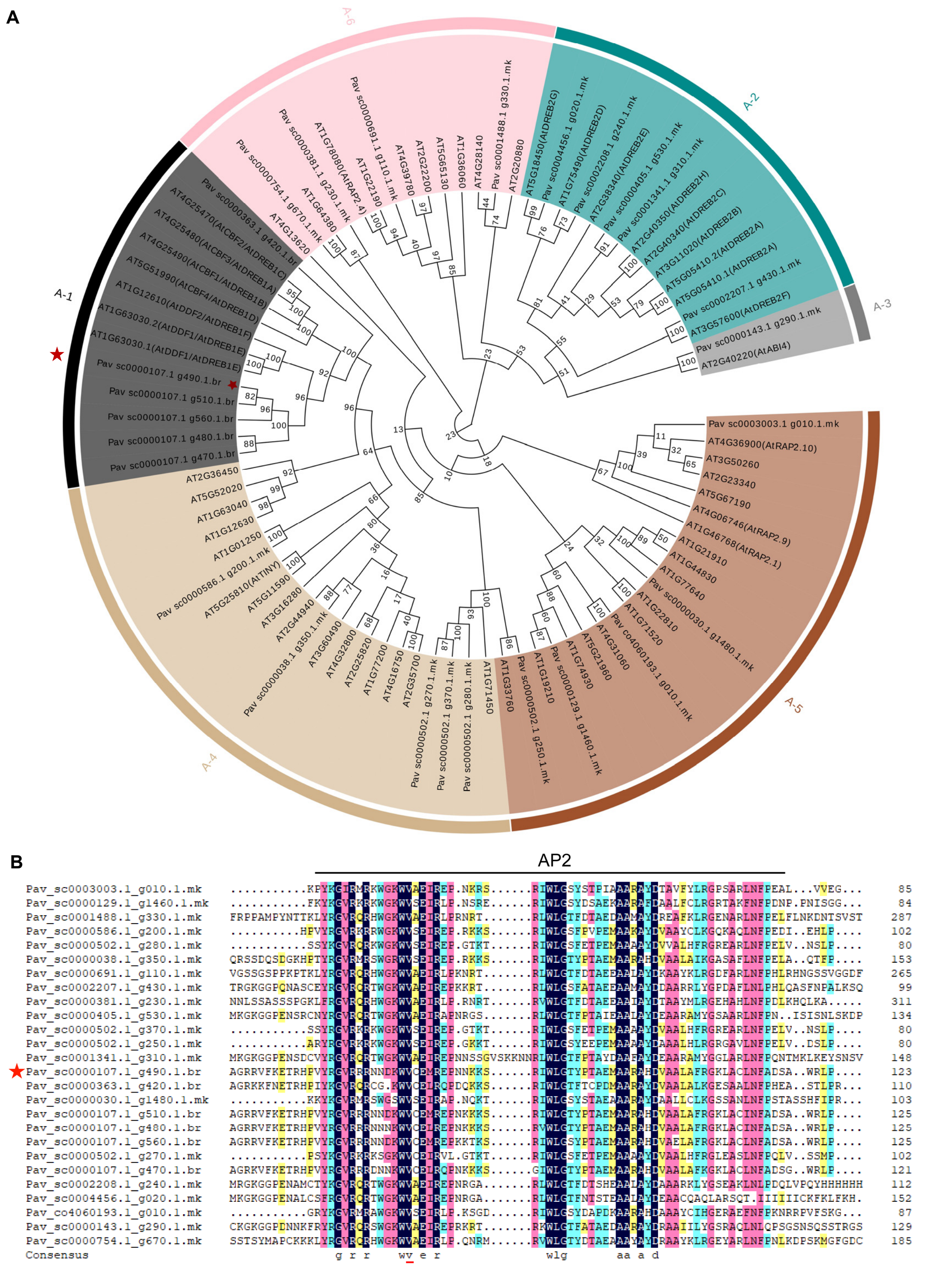

Supplementary Figure S2. Phylogenetic analysis and sequence alignment of the AP2 domains of DREB proteins from sweet cherry. A, Phylogenetic analysis of DREB proteins from sweet cherry (Pav, *Prunus avium*) and Arabidopsis (At, *Arabidopsis thaliana*). Numbers represent bootstrap values from 1,000 replicates. **B**, Multiple amino acid sequence alignment of the AP2 domains of the DREB proteins from sweet cherry. Val-14 in the AP2 domain is underlined in red.

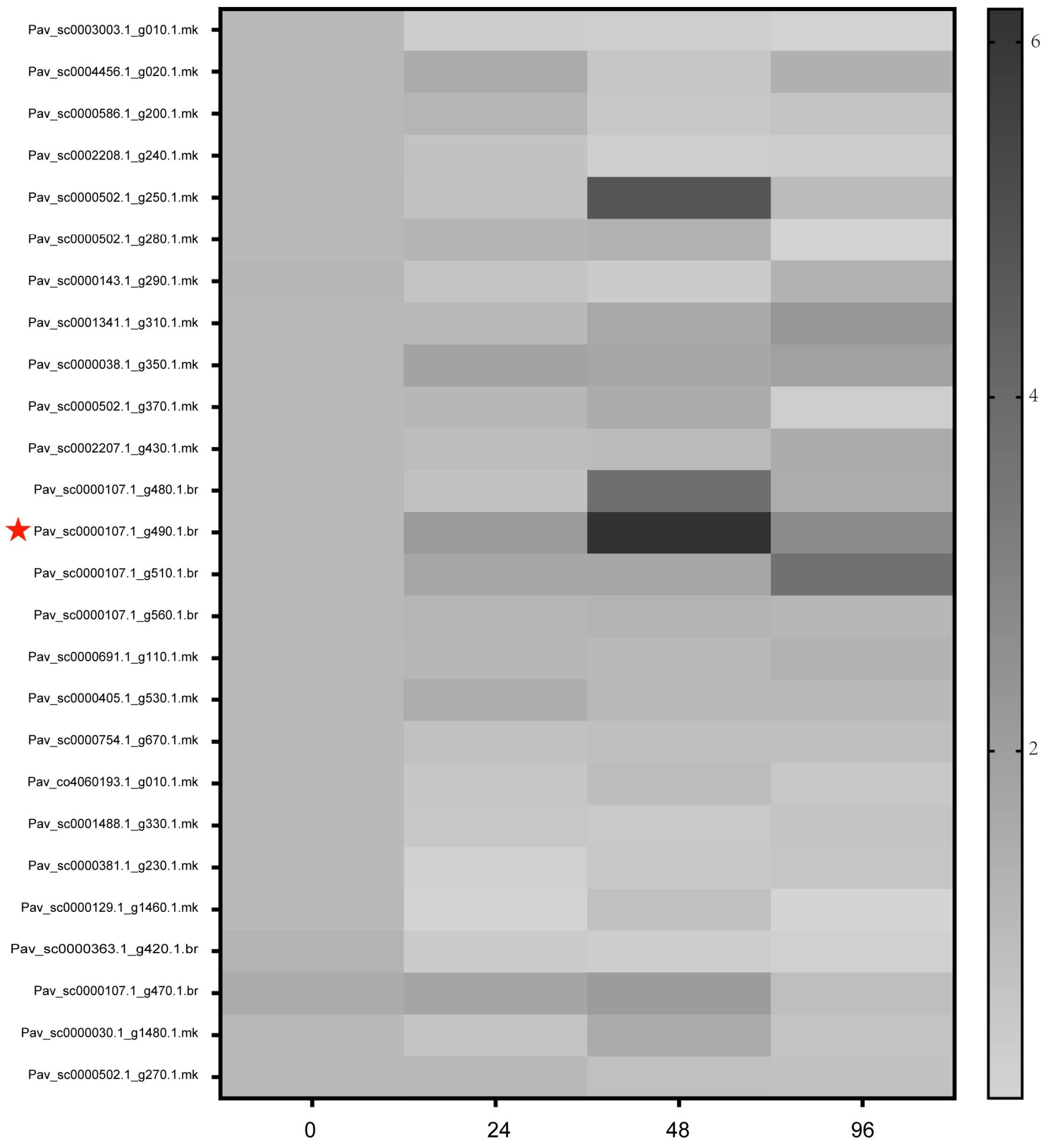

Supplementary Figure S3. *PavDREB* transcript levels in sweet cherry fruits in response to light exposure. The heatmap shows *PavDREB* transcript levels in response to light treatment for 0, 24, 48, or 96 h. Transcript levels were obtained by RT-qPCR analysis. *PavACTIN1* was used as an internal control.

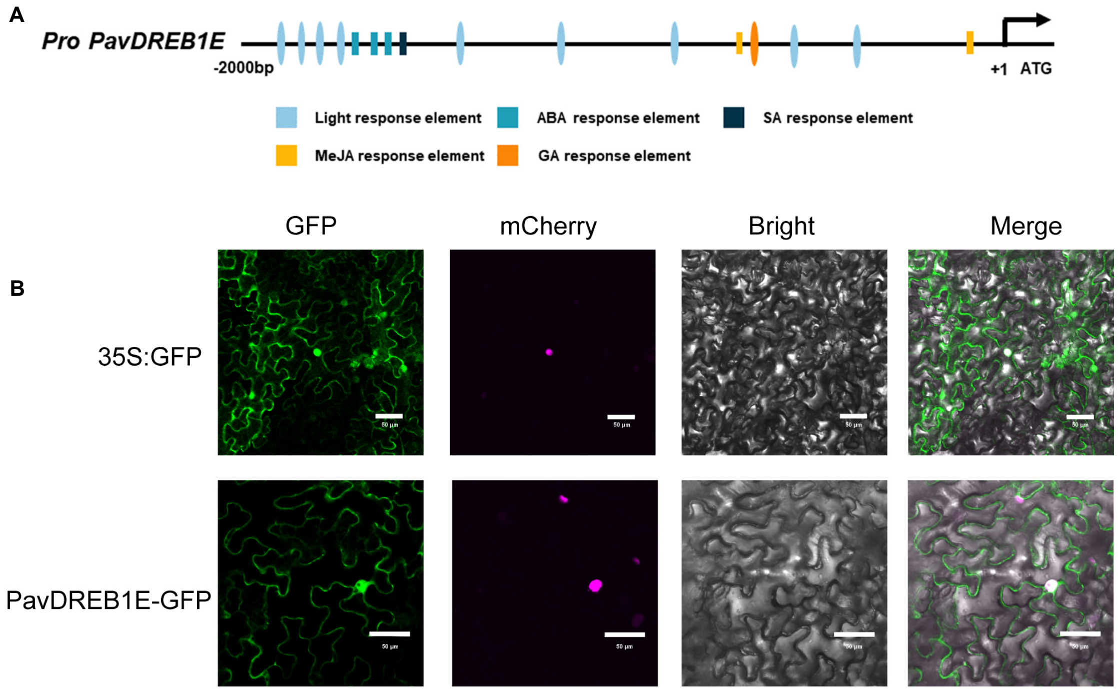

Supplementary Figure S4. Motif analysis of the *PavDREB1E* promoter and subcellular localization of PavDREB1E. A, *Cis*-element in the *PavDREB1E* promoter. Colored boxes represent different *cis*-elements. **B,** PavDREB1E is predominantly distributed in the nucleus. The *35S:PavDREB1E-GFP* construct was infiltrated into the leaves of *Nicotiana benthamiana* plants; the GFP signal was observed 48 h later. The *35S:GFP* construct served as the control. The *NF-YA4-mCherry* construct was co-infiltrated as a nucleus marker. Scale bars, 50 µm.

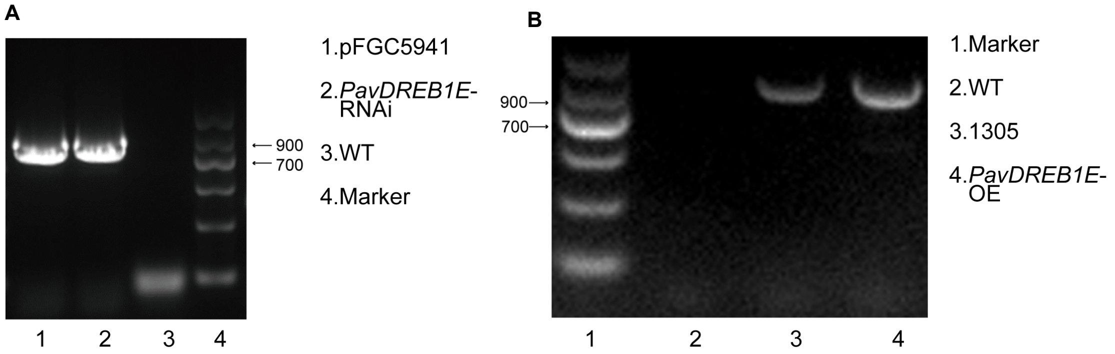

Supplementary Figure S5. Confirmation of the transformation of bicolored sweet cherry fruits. A, Amplification of an 888-bp PCR product using genomic DNA extracted from transgenic fruits as a template and a primer pair specific for *CHSA*. **B,** Amplification of a 935-bp PCR product using genomic DNA extracted from transgenic fruits as a template and a primer pair specific for the CaMV 35S promoter.

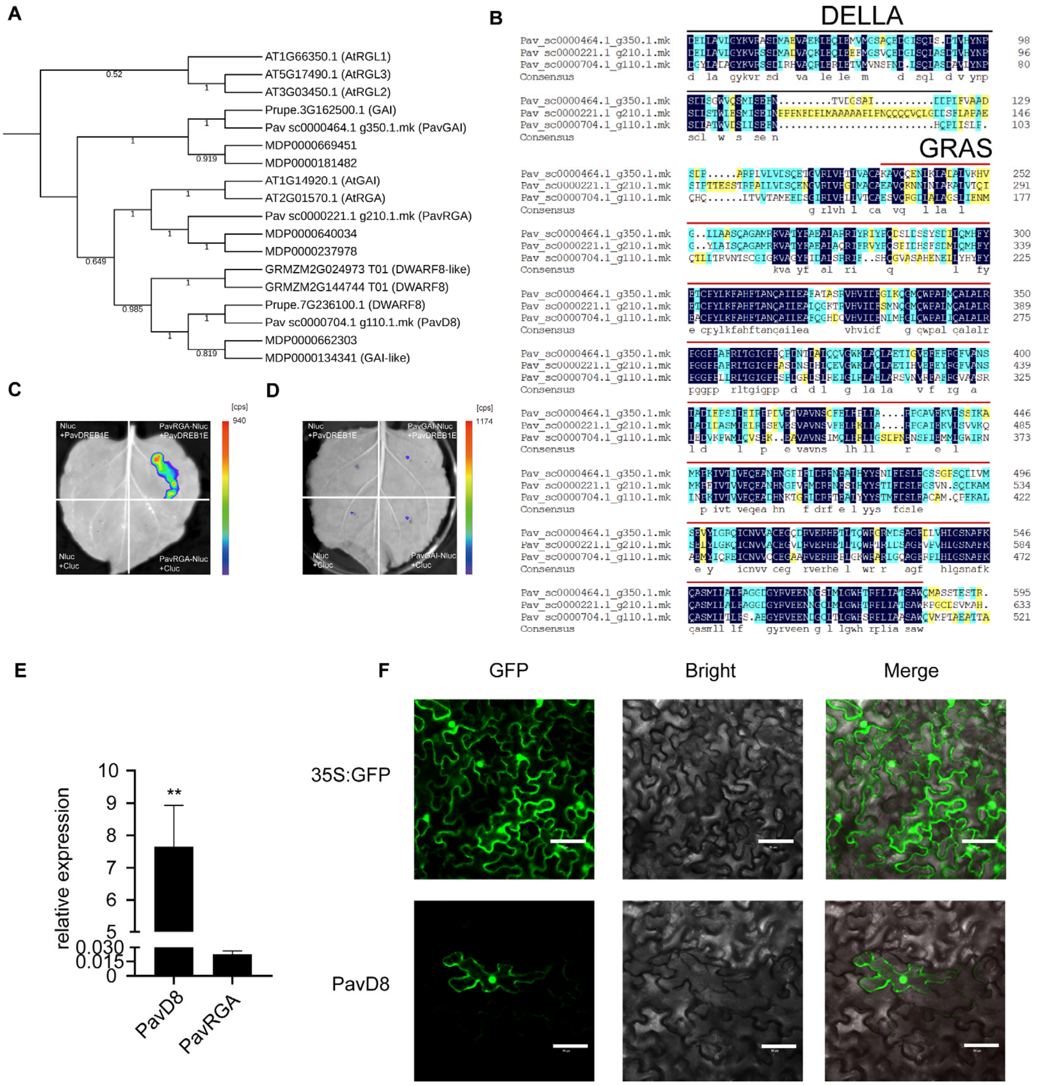

Supplementary Figure S6. Identification and analysis of DELLAs from sweet cherry. A, Phylogenetic analysis of DELLAs in sweet cherry (*Prunus avium*), peach (*Prunus persica*), apple (*Malus domestica*), Arabidopsis (*Arabidopsis thaliana*), and maize (*Zea mays*). Numbers represent bootstrap values from 1,000 replicates. **B,** Multiple amino acid sequence alignment of the DELLA and GRAS domains of sweet cherry DELLA proteins. **C and D,** Analysis of the interaction between PavDREB1E and PavRGA (c) and PavGAI (d) using a luciferase complementation imaging assay. **E,** Relative expression levels of *PavD8* and *PavRGA* in sweet cherry fruits. *PavACTIN1* was used as an internal control. Values are means ± standard deviation (SD) from 3 replicates. The significance of differences was determined using Student's *t*-test; $^{**}P < 0.01$. **F,** Localization analysis of PavD8. A *35S:PavD8-GFP* construct was infiltrated into *N. benthamiana* leaves; the GFP signal was observed 48 h later. Scale bars, 50 µm.

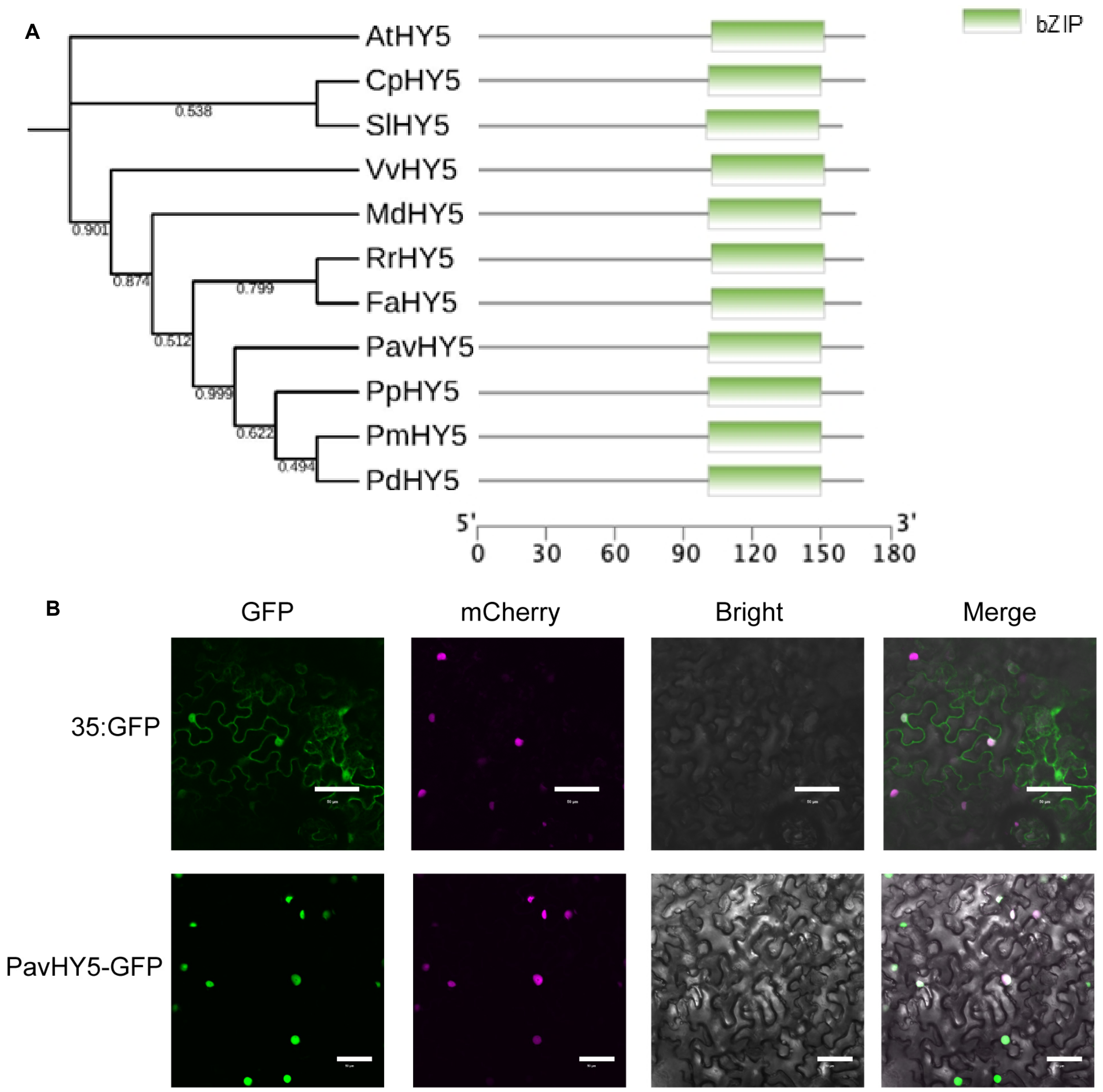

Supplementary Figure S7. Phylogenetic analysis and subcellular localization of PavHY5. A, Phylogenetic relationship and conserved domains of HY5 from sweet cherry (*Prunus avium*), grape (*Vitis vinifera*), Arabidopsis (*Arabidopsis thaliana*), almond (*Prunus dulcis*), Japanese apricot (*Prunus mume*), peach (*Prunus persica*), Asian pear (*Pyrus pyrifolia*), strawberry (*Fragaria* × *ananassa*), apple (*Malus domestica*), papaya (*Carica papaya*) and rose (*Rosa rugosa*). The numbers on the phylogenetic tree represent bootstrap support values from 1,000 replicates. Numbers below the bar represent the length in amino acids. **B,** PavHY5 is located in the nucleus. The *35S:PavHY5-GFP* or *35S:GFP* construct was co-infiltrated with *35S:NF-YA4-mCherry* into *N. benthamiana* leaves; GFP and mCherry fluorescence signals were observed 48 h later. Scale bars, 50 µm.

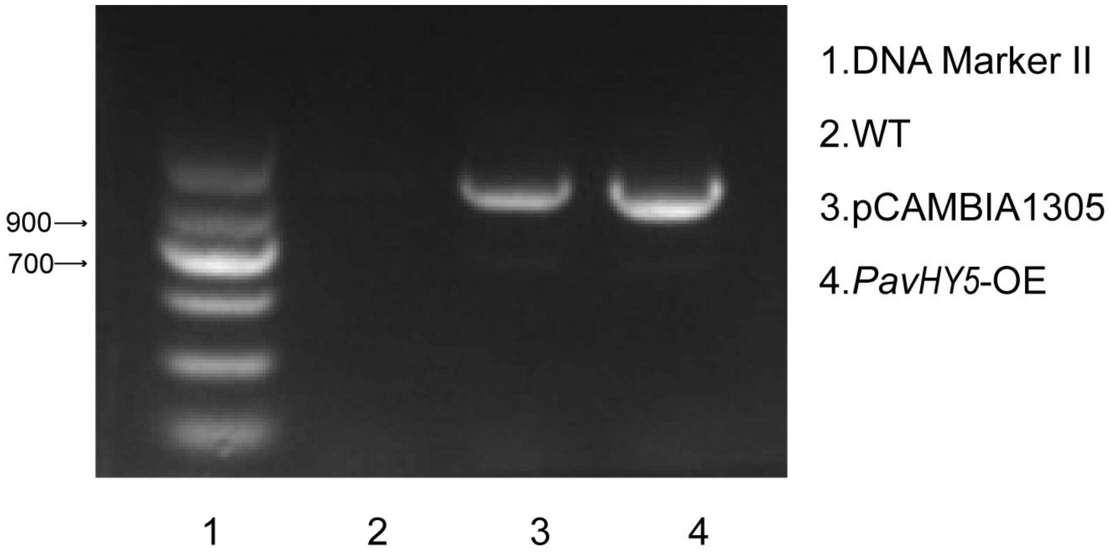

Supplementary Figure S8. Validation of *PavHY5*-OE in bicolored sweet. Amplification of a 935-bp PCR product using genomic DNA extracted from transgenic fruits as a template and a primer pair specific for the CaMV 35S promoter.

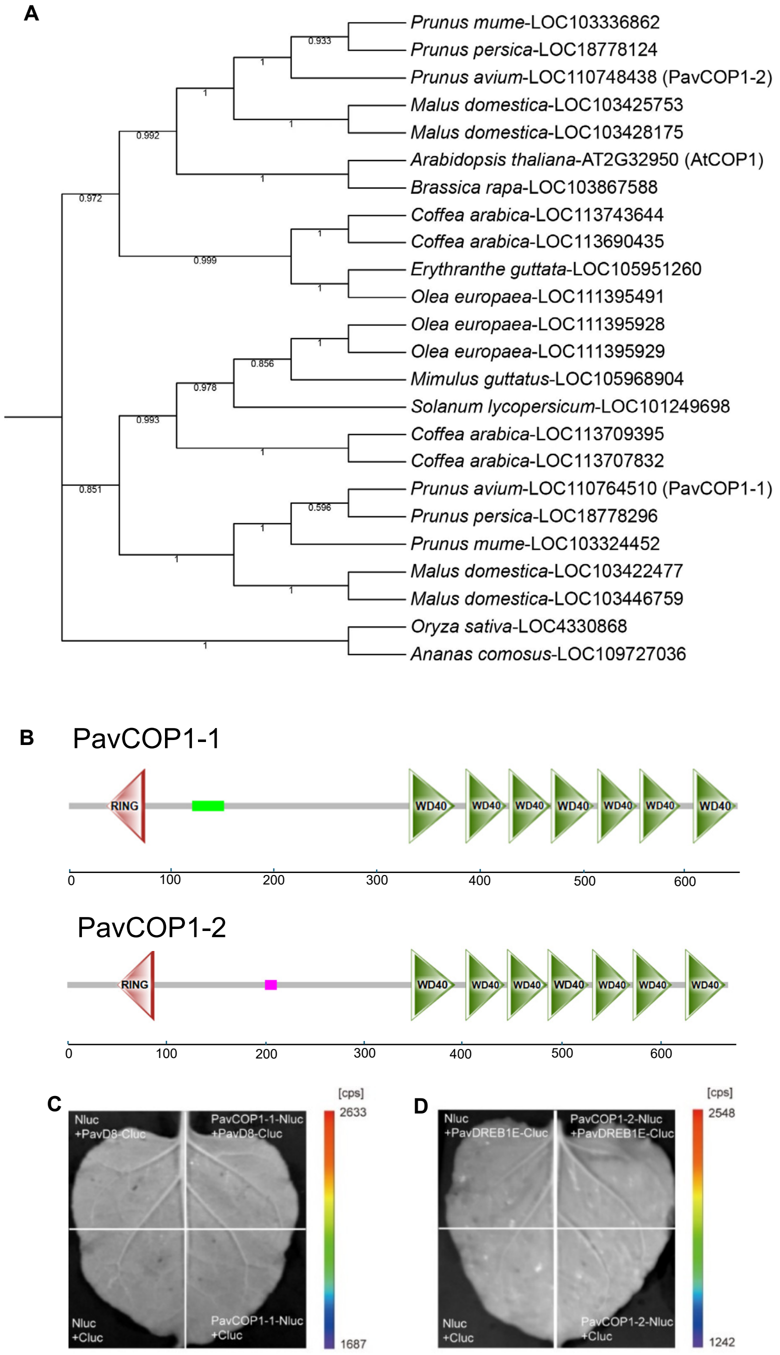

Supplementary Figure S9. Analysis of PavCOP1-1 and PavCOP1-2. A, Phylogenetic analysis of COP1 from sweet cherry (*Prunus avium*) and other species. Numbers represent bootstrap values from 1,000 replicates. **B,** Analysis of the functional conserved domains in the two COP1 genes of sweet cherry (*Prunus avium*). Numbers below the bar represent the length in amino acids. **C,** PavD8 and PavCOP1-1 interact, as determined by a luciferase complementation imaging assay. **D,** Interaction between PavDREB1E and PavCOP1-1, as determined by a luciferase complementation imaging assay.

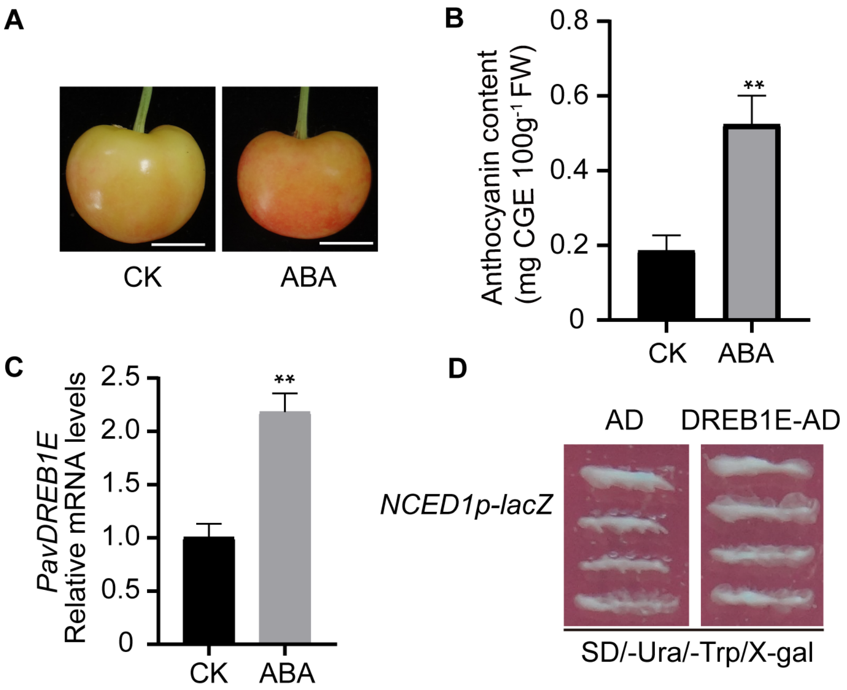

Supplementary Figure S10. Effects of ABA treatment on sweet cherry fruit coloration and *PavDREB1E*. A, Representative photographs of Rainier fruits subjected to ABA treatment. Fruits were treated with H_2O or ABA (100 μM) in the light, control; ABA, Abscisic acid. Scale bar, 1 cm. **B,** Quantification of anthocyanin contents in sweet cherry fruits under various conditions. CGE, cyanidin-3-galactoside equivalents. FW, fresh weight. **C,** Relative expression levels of *PavDREB1E* in response to ABA treatment, as determined by RT-qPCR. *PavACTIN1* served as an internal control. In (**B–C**), values are means ± standard deviation (SD) of three biological replicates (15 fruits per replicate). The significance of differences was assessed using Student's *t*-test (**$P < 0.01$). **D,** Y1H assay showing that PavDREB1E does not interact with the core ABA biosynthesis gene *PavNCED1*.
